# Multicomponent synthesis of artificial nucleases and their RNase and DNase activity

**DOI:** 10.3762/bjoc.7.131

**Published:** 2011-08-19

**Authors:** Anton V Gulevich, Lyudmila S Koroleva, Olga V Morozova, Valentina N Bakhvalova, Vladimir N Silnikov, Valentine G Nenajdenko

**Affiliations:** 1 Department of Chemistry, Moscow State University, 119992, Leninskie Gory, Moscow, Russia; 2 Institute of Chemical Biology and Fundamental Medicine, Siberian Branch of Russian Academy of Sciences, 8 Lavrentyev Ave., 630090 Novosibirsk, Russia; 3 Novosibirsk State University, 2 Pirogova St., 630090 Novosibirsk, Russia; 4 Institute of Systematic and Ecology of Animals, Siberian Branch of Russian Academy of Sciences, 11 Frunze Street, 630091 Novosibirsk, Russia

**Keywords:** DNA, isocyanide, multicomponent reaction, organocatalysis, peptidomimetic, RNA

## Abstract

The synthesis of new, artificial ribonucleases containing two amino acid residues connected by an aliphatic linker has been developed. Target molecules were synthesized via a catalytic three-component Ugi reaction from aliphatic diisocyanides. Preliminary investigations proved unspecific nuclease activity of the new compounds towards single-stranded RNA and double-stranded circular DNA.

## Introduction

RNA cleavage can serve as a molecular tool for biological research [1], as well as for development of anticancer drugs [2–3] and new therapeutics against RNA-containing viruses. Recently, a number of synthetic RNA-cleaving molecules (artificial ribonucleases) had been developed and tested in vitro [4–11]. Among numerous artificial ribonucleases, peptidomimetics showed evident advantages due to their lower cytotoxicity and elevated potential penetration into living eukaryotic cells. Moreover, a few dipeptides [12] were shown to induce interferon production, thus providing antivirus defence. Therefore, the development of new peptidomimetics with ribonuclease activity is an important task in organic and biomolecular chemistry. In this paper we present the rational design, multicomponent synthesis, and RT-qPCR quantitation of nuclease activity of novel amino acids derivatives.

## Results and Discussion

**Figure 1:**
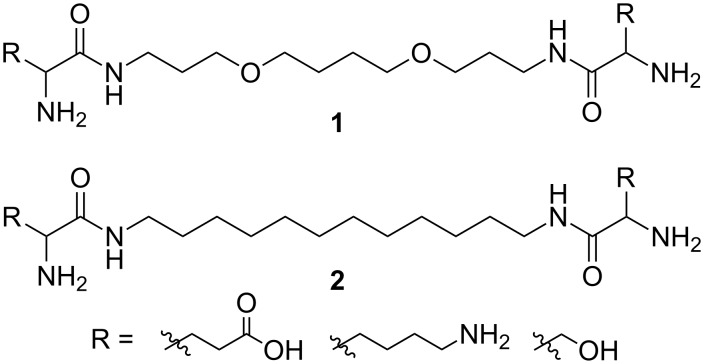
Novel artificial RNases based on amino acids.

Recently, a number of artificial peptide ribonucleases modeling known catalytic centers of natural RNases A and T1 have been described [13–15]. RNA cleavage was shown to be more efficient in the presence of aliphatic hydrophobic linkers [16]. However, the potential role of the alkyl chain of the catalyst remains unclear. Interaction of hydrophobic residues in peptides was suggested to result in formation of RNase mimetics in solution thus enhancing their ribonuclease activity. To prove this suggestion, symmetric aliphatic diamides **1** and **2** containing natural amino acid residues have been synthesized (Figure 1). The compounds showed high ribonuclease activity with model oligoribonucleotides and an HIV-1 recombinant RNA fragment 96 nucleotides long [17].

Previously, compounds **1** and **2** have been synthesized from the corresponding diamines by condensation with protected natural amino acids and subsequent deprotection [18]. This approach is significantly limited by using available natural amino acids (R is a natural amino acid residue). Consequently, the development of new simple, atom-economic methods for the synthesis of this class of potential biologically active compounds is of great importance in bioorganic and medicinal chemistry. The development of multicomponent approaches is especially important because multicomponent reactions (MCR) could be adapted to a high throughput synthesis of libraries of compounds.

**Scheme 1:**
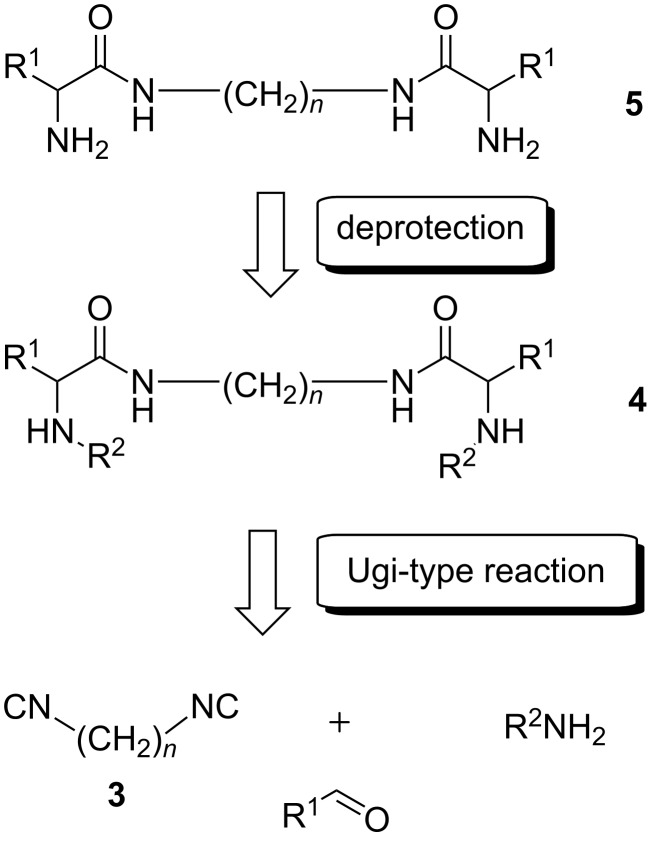
Multicomponent approach to target molecules.

It is known that isocyanide-based MCR are very efficient for synthesis of peptides and peptide molecules [19–24]. We proposed that the desired compounds **5**, containing two amide bonds and variable substituents, can be synthesized by the Ugi reaction with subsequent removal of diamine residue (Scheme 1). Original substrates for the synthesis could be aliphatic diisocyanides **3**, amines (with an easily removable protective group) and aldehydes. We used an organocatalytic three-component modification of the Ugi reaction, recently developed by List et al. [25]. The reaction results in diamines **4**, thus avoiding the acid residue removal stage.

**Scheme 2:**
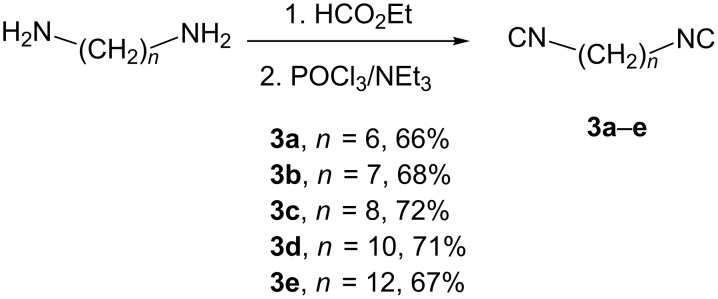
Synthesis of starting diisocyanides **3a**–**3e**.

The starting diisocyanides **3** were obtained in good overall yields from commercially available diamines containing 6, 7, 8, 10 or 12 carbon atoms by the standard formylation–dehydration protocol (Scheme 2).

**Scheme 3:**
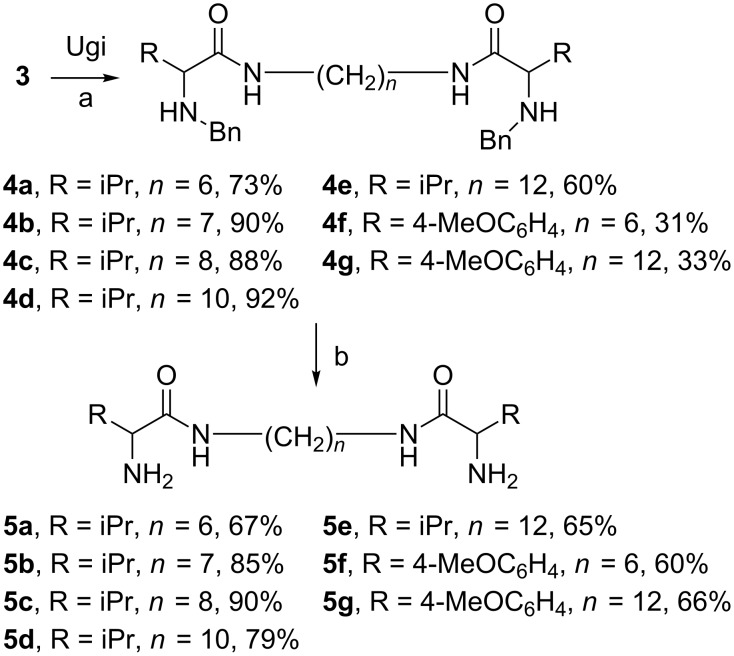
Synthesis of new aRNAses. Conditions: a. RCHO (3 eqiuv), BnNH_2_ (3 equiv), PhP(OH)_2_ (1 equiv), r.t.; b. Pd/C, 5%, HCOONH_4_, MeOH/H_2_O, reflux.

We found that these diisocyanides **3** participate successfully in the catalytic three-component reaction via a modified List procedure [25]. Diamides **4** with benzyl protective groups were synthesized in moderate to good yields under mild conditions. There is no obvious dependence of yield on the length of the carbon chain in **3**; aliphatic aldehydes gave better results in comparison to aromatic aldehydes (Scheme 3). Obviously, the suggested approach is an efficient and short method to form the skeleton of the target diamides. The benzyl groups can be easily removed from compounds **4** by the standard hydrogenolysis procedure. For example, using Pd/C as catalyst we obtained the target peptidomimetics **5** in up to 90% yield. Thus, we synthesized a number of racemic peptidomimetics **4** and **5**, containing aliphatic or aromatic groups as well as various aliphatic linkers. With these diamides in hand we began the investigation of their biological activity.

Currently, real-time PCR is the better method for the quantitation of the target nucleic acids because of its high specificity and sensitivity of up to a few genome equivalents in a complex mixture [26]*.* In the present work, ribonuclease activity of the new synthesized compounds was studied in vitro by cleavage of the total cellular and the tick-borne encephalitis virus (TBEV) full-length genomic RNA isolated from infected mouse brain, with subsequent detection by RT-qPCR (TBEV is a human pathogenic member of the *Flavivirudae* family of RNA-containing viruses of positive polarity).

**Figure 2:**
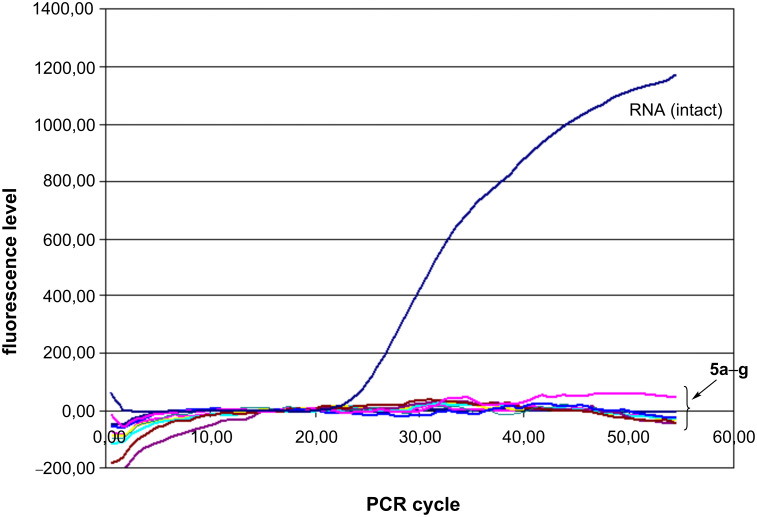
Results of RT-qPCR of the TBEV RNA cleavage products in presence of 2.5 mM peptidomimetics **5a–g** at 37 °C for 2 hours in H_2_O.

Complete cleavage of 2 µg of cellular RNA including 10^5^ genome equivalents of the TBEV full-length RNA was observed after incubation of the total RNA from the virus-infected mouse brain with 2.5 mM aqueous solutions of peptidomimetics **5a**–**g** for 2 hours at 37 °C. Denaturating electrophoresis in SDS-agarose gel revealed complete cleavage of the total RNA (Supporting Information File 1, Figure S1) and RT-real time PCR showed complete destruction of the TBEV RNA (Figure 2).

Compounds **5e** and **5g**, the most hydrophobic among synthesized substances, might potentially penetrate through cellular or viral membranes and therefore the dependence of RNA cleavage on the concentration of the peptidomimetics was studied in detail. The concentration of compounds **5e** and **5g**, optimal for RNA cleavage, was determined by varying the concentration from 2.5 × 10^−3^ to 2.5 × 10^−7^ M (Supporting Information File 1, Figure S2 A and B). Dependence of optimal RNA cleavage on the concentration of peptidomimetics was not evident: Complete RNA cleavage of 2 μg RNA was observed at a concentration of 2.5 mM for both **5e** with an aliphatic substituent and **5g** with an aromatic one.

**Figure 3:**
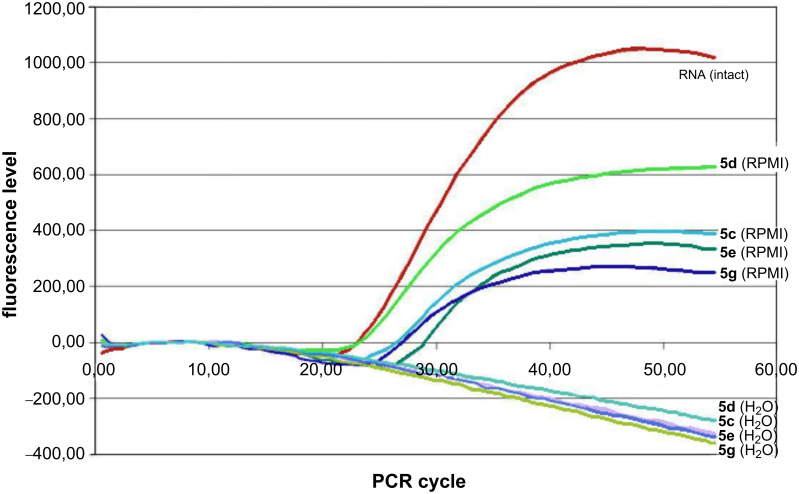
Results of RT-qPCR of the TBEV RNA cleavage products in the presence of 2.5 mM peptidomimetics after incubation in H_2_O and in cultural medium RPMI 1640.

**Figure 4:**
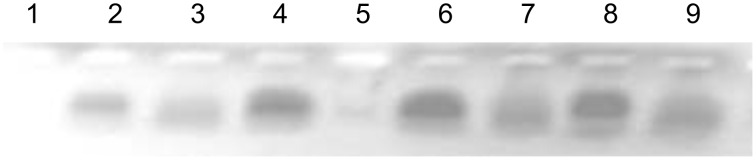
Results of electrophoresis in 2% TBE-agarose gel with ethidium bromide of RT-qPCR products from Figure 3. (Cleavage of total RNA from 10% brain suspensions from ICR mice infected with TBEV). Lanes: 1 - negative control; 2 and 3 - compound **5e** incubated with RNA in RPMI 1640 and in H_2_O, respectively; 4 and 5 - compound **5g** incubated with RNA in RPMI 1640 and in H_2_O, respectively; 6 and 7 - compound **5c** incubated with RNA in RPMI 1640 and in H_2_O, respectively; 8 and 9 - compound **5d** incubated with RNA in RPMI 1640 and in H_2_O, respectively.

Ribonuclease activity of the compounds **5c–e** and **5g** at 2.5 mM concentration was assayed in cultural medium RPMI 1640 and compared cleavage in H_2_O. All artificial RNases cleaved RNA more efficiently in water than in RPMI 1640 (Figure 3 and Figure 4). Results of RT-real time PCR (Figure 3) and electrophoresis RT-qPCR products in 2% TBE-agarose gel (Figure 4) showed varying degrees of destruction of the TBEV RNA, respectively.

To analyze DNase activity of the novel compounds, both double-stranded circular recombinant plasmid DNA with cloned full-length TBEV copy of genome and single-stranded cDNA after reverse transcription of the TBEV RNA from the infected mouse brain with random N_6_ primer was used. No destruction of single-stranded cDNA was observed after incubation with 2.5 mM solutions of compounds **5e** or **5g** for 2 hours at 37 °C (Supporting Information File 1, Figure S3). However, these compounds could partly cleave double-stranded plasmid DNA (Supporting Information File 1, Figure S4).

Generally, all synthesized compounds were shown to be able to cleave completely single-stranded RNA but not single-stranded cDNA or hybrid of RNA with cDNA after reverse transcription irrespective of the structures of their substituents and the length of polymethylene linkers. Double-stranded circular plasmid DNA was partially destroyed possibly because of single-stranded DNA breaks. The mechanism has been previously shown for several artificial metal-free [27] and metal-dependent nucleases [28]. A further study of the TBEV RNA cleavage, both in extracellular virions and within infected cells as well as specific cleavage of only viral RNA, is required. Further investigations of developed artificial RNases are in progress.

## Conclusion

New artificial nucleases based on diamides containing two amino acid residues connected by aliphatic linkers were synthesized by a catalytic three-component Ugi-type reaction and subsequent deprotection. To quantitative cleavage of any nucleic acids, including single-stranded RNA and cDNA as well as double-stranded circular plasmid DNA, high-throughput RT-qPCR was developed and used. All synthesized compounds were shown to be able to cleave completely single-stranded RNA but not single-stranded cDNA or hybrid of RNA with cDNA after reverse transcription irrespective of the structure of their substituents and the length of the polymethylene linker.

## Experimental

### General procedure for the synthesis of diisocyanides **3**

A solution of the corresponding diamine (0.1 mol) in ethyl formate (100 mL) was heated under reflux for 5 h. The reaction mixture was concentrated in vacuo. The resulting formamide (without additional purification) was suspended in anhydrous dichloromethane (200 mL) and triethylamine (51 g, 0.5 mol) added. The mixture was cooled to 0 °C and POCl_3_ (0.21 mol, 32 g) added dropwise at such a rate that the reaction temperature remained below 0 °C. The mixture was stirred for 2 h. The reaction mixture was poured into ice–water (500 mL) containing K_2_CO_3_ (100 g) maintaining the temperature below 25 °C. The resulting emulsion was stirred for 1 h at rt. The organic layer was separated, the aqueous layer extracted with CH_2_Cl_2_ (2 × 50 mL), and the combined organic layers were dried (K_2_CO_3_), purified by flash chromatography and concentrated in vacuo*.* The diisocyanide was obtained as a dark oil.

**1,6-Diisocyanohexane (3a):** Yield 66%, dark oil, *R*_f_ 0.8 (hexane/EtOAc 2:1); ^1^H NMR (400 MHz, CDCl_3_) δ 1.39–1.47 (m, 4H, (C*H_2_*CH_2_CH_2_NC)_2_), 1.55–1.73 (m, 4H, (CH_2_C*H_2_*CH_2_NC)_2_), 3.30–3.40 (m, 4H, (CH_2_CH_2_CH_2_NC)_2_); ^13^C NMR (100 MHz, CDCl_3_) δ 155.6 (t, *J* = 5.9 Hz, NC), 41.1 (t, *J* = 6.6 Hz, (CH_2_CH_2_*C*H_2_NC)_2_), 28.5, 25.2; IR (cm^−1^) 2150 (NC); Anal. calcd for C_8_H_12_N_2_: C, 70.55; H, 8.88; found: C, 70.34; H, 8.62.

### General procedure for the synthesis of **4a**–**g**

The corresponding isocyanide **3** (3 mmol) and phenyl phosphinic acid (3 mmol, 441 mg) were added to a mixture of the aldehyde (9 mmol) and benzylamine (9 mmol, 963 mg) in CH_2_Cl_2_ or MeOH (30 mL). The mixture was stirred for 48 h at rt, the solvent removed in vacuo and the residue purified by column chromatography (hexane/ethyl acetate 1:1). The product (colorless oil or white solid) can be converted into the corresponding hydrochloride by treatment with gaseous HCl in MeOH.

**Compound 4a:** The reaction was carried out in CH_2_Cl_2_, yield 73%; colorless oil; *R*_f_ 0.4 (hexane/EtOAc 1:1); ^1^H NMR (400 MHz, CDCl_3_) δ 0.86 (d, *J* = 7.1 Hz, 6H, 2 × CH_3_), 0.94 (d, *J* = 7.1 Hz, 6H, 2 × CH_3_), 1.29–1.36 (m, 4H, (C*H_2_*CH_2_CH_2_NHCO)_2_), 1.44–1.52 (m, 4H, (CH_2_C*H_2_*CH_2_NHCO)_2_), 1.6 (br s, 2H, 2 × NH), 2.06–2.15 (m, 2H, 2 × C*H*(CH_3_)_2_), 2.95 (d, *J* = 4.3 Hz, 2H, 2 × C*H*), 3.17–3.30 (m, 4H, (CH_2_CH_2_C*H_2_*NHCO)_2_), 3.68 (AB-system, *J* = 13.1 Hz, 4H, 2 × C*H_2_*Ph), 7.20–7.35 (m, 12H, Ph, 2 × NH); ^13^C NMR (100 MHz, CDCl_3_) δ 173.3, 139.6, 128.6, 128.1, 127.3, 67.9, 53.5, 38.6, 31.2, 29.7, 26.5, 19.6, 17.7; IR (cm^−1^) 1640 (*CO*NH), 3300 (br, CO*NH*); ESI-MS (*m*/*z*): [M + H]^+^ calcd for C_30_H_46_N_4_O_2_, 495.3621; found, 495.3698.

### Cleavage of the benzyl group

A solution of HCOONH_4_ (1 g in 5 mL H_2_O) was added to a solution of the corresponding amide **4** (1 mmol) in 10 mL of MeOH. The catalyst, Pd/C, (100 mg , 5%) was added and the mixture heated under reflux for 5 h. The mixture was concentrated and treated with aqueous K_2_CO_3_. The product was extracted with CH_2_Cl_2_ (3 × 30 mL), the organic layer dried (K_2_CO_3_) and concentrated in vacuo. The residue was purified by column chromatography (CH_2_Cl_2_/MeOH 10:1). The resulting product (colorless oil or white solid) can be converted into corresponding hydrochloride by treatment with gaseous HCl in MeOH.

**Compound 5a**: Yield 67%; colorless oil; *R*_f_ 0.6 (CH_3_CN/EtOH/NH_3_ 80:12:8); ^1^H NMR (400 MHz, CDCl_3_) δ 0.81 (d, *J* = 7.1 Hz, 6H, 2 × CH_3_), 0.97 (d, *J* = 7.1 Hz, 6H, 2 × CH_3_), 1.30–1.40 (m, 4H, (C*H_2_*CH_2_CH_2_NHCO)_2_), 1.44–1.52 (m, 4H, (CH_2_C*H_2_*CH_2_NHCO)_2_), 2.26–2.36 (m, 2H, 2 × C*H*(CH_3_)_2_), 3.17–3.30 (m, 6H, 2 × C*H*, (CH_2_CH_2_C*H_2_*NHCO)_2_), 7.26–7.33 (m, 2H, 2 × NH); ^13^C NMR (100 MHz, CDCl_3_) δ 174.3, 60.2, 38.6, 31.2, 29.7, 26.5, 19.6, 17.7; IR (cm^−1^) 1638 (*CO*NH), 3290 (br, NH, NH_2_); ESI-MS (*m*/*z*): [M + H]^+^ calcd for C_16_H_34_N_4_O_2_, 314.2682; found, 314.2670.

## Supporting Information

File 1 General information, procedures, spectral data of all compounds, results of bioassay, and copies of selected NMR spectra.
