# Supplementary material for: Multicomponent synthesis of artificial nucleases and their RNase and DNase activity
Source: Beilstein J Org Chem. 2011 Aug 19;7:1135–40. doi: 10.3762/bjoc.7.131 (PMC3170195; doi:10.3762/bjoc.7.131)
Supplement: File 1 — General information, procedures, spectral data of all compounds, results of bioassay, and copies of selected NMR spectra. [file Beilstein_J_Org_Chem-07-1135-s001.pdf]

Supporting Information

for

Multicomponent synthesis of artificial nucleases and their RNase and DNase activity

Anton V. Gulevich[1], Lyudmila S. Koroleva[2,3], Olga V. Morozova[2], Valentina N. Bakhvalova[4], Vladimir N. Silnikov[*2] and Valentine G. Nenajdenko[*1]

Address: [1]Department of Chemistry, Moscow State University, 119992, Leninskie Gory, Moscow, Russia, [2]Institute of Chemical Biology and Fundamental Medicine, Siberian Branch of Russian Academy of Sciences,8 Lavrentyev Ave., 630090 Novosibirsk, Russia, [3]Novosibirsk State University, 2 Pirogova St., 630090 Novosibirsk, Russia and [4]Institute of Systematic and Ecology of Animals, Siberian Branch of Russian Academy of Sciences, 11 Frunze Street, 630091 Novosibirsk, Russia

Email: Valentine G. Nenajdenko[*] - nen@acylium.chem.msu.ru; Vladimir N. Silnikov[*] - Silnik@niboch.nsc.ru

[*]Corresponding author

General information, procedures, spectral data of all compounds, results of bioassay, and copies of selected NMR spectra.

Content

1. General Information 2
2. Experimental details 2
   2.1. General Procedure for the synthesis of diisocyanides 3 2
   2.2. General Procedure for the synthesis of 4a–g: 4
   2.3. Cleavage of the benzyl group 6
3. Investigation of Biological Activity 9
   3.1. RNA isolation 9
   3.2. Cleavage of nucleic acids 9
   3.3. Quantitation of the TBEV RNA, cDNA and plasmid DNA by reverse transcription with subsequent real-time PCR 9
4. Selected NMR Spectra 14

1. General Information

^{1}H and ^{13}C NMR spectra were recorded in deuterated solvents on a Bruker Avance 400 MHz spectrometer. Chemical shifts are reported in parts per million (ppm) or δ values downfield from TMS as internal standard. Residual solvent peaks were used as internal references: $CDCl_3$ at 7.25 and 77.00 ppm. Analytical thin layer chromatography (TLC) was performed on 25 DC-Alufolien Kieselgel 60 F254 (Merck). Visualization was by UV light and or by spraying with aqueous potassium permanganate. Column chromatography was performed with Fluka Silica gel 60 (0.063–0.200 mm). Infrared (IR) spectra were recorded on a Nicolet IR2000 (Thermo Scientific). High-resolution mass spectra (HRMS) were measured on a Micr OTOF II (Bruker Daltonics) spectrometer. Commercially available reagents and solvents were used without special purification.

2. Experimental details

2.1. General Procedure for the synthesis of diisocyanides 3

A solution of corresponding diamine (0.1 mol) in ethylformate (100 ml) was heated under reflux for 5 h. The reaction mixture was concentrated in vacuo and the resulting formamide used in the next step without further purification. Formamide (≈0.1 mol) was suspended in anhydrous dichloromethane (200 ml) and triethylamine (51 g, 0.5 mol) added. The mixture was cooled to 0 °C and $POCl_3$ (0.21 mol, 32 g) added dropwise at such a rate that the reaction temperature remained below 0 °C. The mixture was stirred for 2 h. The reaction mixture was poured into of ice-water (500 mL) containing K_2CO_3 (100 g) maintaing the temperature below 25°C. The resulting emulsion was stirred for 1 h at room temperature. The organic layer was separated, the aqueous layer extracted with CH_2Cl_2 (2x50 ml), and the combined organic layers were dried (K_2CO_3), purified by flash chromatography (CH_2Cl_2-hexanes) and concentrated in vacuo. The diisocyanide was obtained as dark oil.

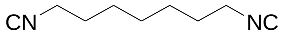

3b

1,7-Diisocyanoheptane 3b: yield 68%, dark oil, R_f (hexane/EtOAc 2/1) 0.8. IR (cm^{-1}) 2152 (NC). ^1H NMR ($CDCl_3$, 400 MHz): 1.31-1.50 (m, 6H, $C\mathit{H_2}(C\mathit{H_2}CH_2CH_2NC)_2$), 1.63-1.75 (m, 4H, $CH_2(CH_2C\mathit{H_2}CH_2NC)_2$), 3.35-3.40 (m, 4H, $CH_2(CH_2CH_2C\mathit{H_2}NC)_2$). ^{13}C NMR ($CDCl_3$, 100 MHz): 155.8 (t, J = 5.9 Hz, NC), 41.4 (t, J = 6.6 Hz, $\mathit{C}H_2NC$), 28.8, 27.8, 26.0. Found, %: C, 72.30; H, 9.98. $C_9H_{14}N_2$. Calculated, %: C, 71.96; H, 9.39.

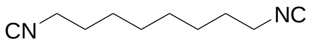

3c

1,8-Diisocyanooctane 3c: yield 72%, dark oil, R_f (hexane/EtOAc 2/1) 0.8. IR (cm^{-1}) 2149 (NC). ^1H NMR ($CDCl_3$, 400 MHz): 1.20-1.37 (m, 8H, $(C\mathit{H_2}C\mathit{H_2}CH_2CH_2NC)_2$), 1.48-1.65 (m, 4H, $(CH_2CH_2C\mathit{H_2}CH_2NC)_2$), 3.25-3.35 (m, 4H, $(CH_2CH_2\ CH_2C\mathit{H_2}NC)_2$). ^{13}C NMR ($CDCl_3$, 100 MHz): 155.6 (t, J 5.1 Hz, NC), 41.5 (t, J = 6.6 Hz, $\mathit{C}H_2NC$), 29.2, 28.6, 26.1. Found, %: C, 73.15; H, 9.77. $C_{10}H_{16}N_2$. Calculated, %: C, 73.13; H, 9.82.

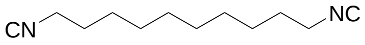

3d

1,10-Diisocyanodecane 3d: yield 71%, dark oil, R_f (hexane/EtOAc 2/1) 0.8. IR (cm^{-1}) 2150 (NC). ^1H NMR ($CDCl_3$, 400 MHz): 1.26-1.33 (m, 8H, $(C\mathit{H_2}C\mathit{H_2}CH_2CH_2CH_2NC)_2$), 1.37-1.48 (m, 4H, $(CH_2CH_2C\mathit{H_2}CH_2CH_2NC)_2$), 1.60-1.72 (m, 4H, $(CH_2CH_2CH_2C\mathit{H_2}CH_2NC)_2$), 3.33-3.40 (m, 4H, $(CH_2CH_2CH_2CH_2C\mathit{H_2}NC)_2$). ^{13}C NMR ($CDCl_3$, 100 MHz): 155.5 (t, J = 5.9 Hz, NC), 41.5 (t, J = 6.6 Hz, $\mathit{C}H_2NC$), 29.1, 29.0, 28.6, 26.2. Found, %: C, 75.20; H, 10.99. $C_{12}H_{20}N_2$. Calculated, %: C, 74.95 H, 10.48.

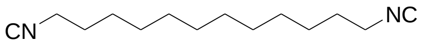

3e

1,12-Diisocyanododecane 3e: yield 67%, dark oil, R_f (hexane/EtOAc 2/1) 0.8. IR (cm^{-1}) 2147 (NC). ^1H NMR ($CDCl_3$, 400 MHz): 1.22-1.32 (m, 12H, $(C\mathit{H_2}C\mathit{H_2}C\mathit{H_2}CH_2CH_2CH_2NC)_2$), 1.36-1.45 (m, 4H, $(CH_2CH_2CH_2C\mathit{H_2}CH_2CH_2NC)_2$), 1.58-1.71 (m, 4H, $(CH_2CH_2CH_2CH_2C\mathit{H_2}CH_2NC)_2$), 3.35 (tt, J = 1.8, 6.8 Hz, 4H, $(CH_2CH_2CH_2CH_2CH_2C\mathit{H_2}NC)_2$). ^{13}C NMR ($CDCl_3$, 100 MHz): 155.4 (t, J = 5.1 Hz, NC), 41.5 (t, J = 5.9 Hz, $\mathit{C}H_2NC$), 29.3, 29.2, 29.0, 28.6, 26.2. Found, %: C, 76.40; H, 11.40. $C_{14}H_{24}N_2$. Calculated, %: C, 76.31 H, 10.98.

2.2. *General Procedure for the synthesis of 4a–g:*

The corresponding diisonitrile (3 mmol) and phenyl phosphinic acid (3 mmol, 441 mg) were added to a mixture of the aldehyde (9 mmol) and benzylamine (9 mmol, 963 mg) in CH_2Cl_2 or MeOH (30 ml). The mixture was stirred for 48 h at room temperature, the solvent removed in vacuo and the residue purified by column chromatography (hexane:ethyl acetate 1:1). The obtained product (colorless oil or white solid) can be converted into corresponding hydrochloride by treatment with gas HCl in MeOH. The hydrochlorides were obtained as white solids.

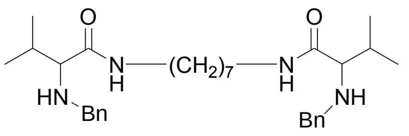

4b

4b: reaction solvent: CH_2Cl_2, yield 90%, colorless oil, R_f (hexane/EtOAc 1/1) 0.4. ^{1}H NMR (400 MHz, $CDCl_3$): 0.86 (d, J = 7.1 Hz, 6H, 2*CH_3), 0.94 (d, J = 7.1 Hz, 6H, 2*CH_3), 1.24-1.35 (m, 6H, $CH_2(CH_2CH_2CH_2NHCO)_2$), 1.40-1.52 (m, 4H, $CH_2(CH_2CH_2CH_2NHCO)_2$), 1.7 (br. s, 2H, 2*NH), 2.05-2.15 (m, 2H, 2*C*H*$(CH_3)_2$), 2.94 (m, J = 4.6 Hz, 2*C*H*), 3.17-3.30 (m, 4H, $CH_2(CH_2CH_2CH_2NHCO)_2$), 3.67 (AB-system, J = 13.1 Hz, 4H, 2*CH_2Ar), 7.20-7.35 (m, 12H, Ar, 2*NH). IR (cm^{-1}) 1635 (*CO*NH), 3300 (br. NH, CO*NH*). ESI-MS (m/z): calcd. for $C_{31}H_{48}N_4O_2$ [M+H$^+$] 509.3777, found 509.3766.

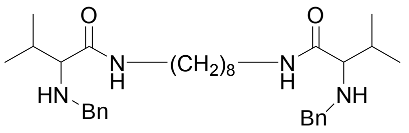

4c

4c: reaction solvent: CH_2Cl_2, yield 88%, colorless oil, R_f (hexane/EtOAc 1/1) 0.4. ^{1}H NMR (400 MHz, $CDCl_3$): 0.86 (d, J = 6.8 Hz, 6H, 2*CH_3), 0.94 (d, J = 6.8 Hz, 6H, 2*CH_3), 1.24-1.35 (m, 8H, $(CH_2CH_2CH_2CH_2NHCO)_2$), 1.40-1.50 (m, 4H, $(CH_2CH_2CH_2CH_2NHCO)_2$), 1.6 (br. s, 2H, 2*NH), 2.05-2.15 (m, 2H, 2*C*H*$(CH_3)_2$), 2.94 (d, J = 4.3 Hz, 2*C*H*), 3.16-3.27 (m, 4H, $CH_2(CH_2CH_2CH_2NHCO)_2$), 3.67 (AB-system, J = 13.1 Hz, 4H, 2*CH_2Ar), 7.15-7.35 (m, 12H, Ar, 2*NH). ^{13}C NMR (100 MHz, $CDCl_3$): 173.3, 139.7, 128.5, 128.1, 127.3, 67.0, 53.5, 38.8, 31.3, 29.7, 29.1, 26.9, 19.6, 17.7. IR (cm^{-1}) 1640 (*CO*NH), 3310 (br. NH, CO*NH*). ESI-MS (m/z): calculated $C_{32}H_{50}N_4O_2$ [M+H$^+$] 523.3934, found 523.4007.

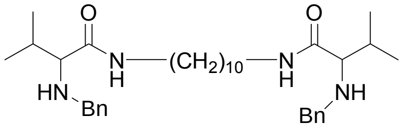

4d

4d: reaction solvent: CH_2Cl_2, yield 92%, colorless oil, R_f (hexane/EtOAc 1/1) 0.4. ^{1}H NMR (400 MHz, $CDCl_3$): 0.86 (d, J = 6.8 Hz, 6H, 2*CH_3), 0.93 (d, J = 6.8 Hz, 6H, 2*CH_3), 1.20-1.32 (m, 12H, $(C\mathit{H_2}C\mathit{H_2}C\mathit{H_2}CH_2CH_2NHCO)_2$), 1.40-1.50 (m, 4H, $(CH_2CH_2CH_2C\mathit{H_2}CH_2NHCO)_2$), 1.5 (br. s, 2H, 2*NH), 2.03-2.16 (m, 2H, 2*$C\mathit{H}(CH_3)_2$), 2.93 (d, J = 4.3 Hz, 2*C*H*), 3.16-3.27 (m, 4H, $(CH_2CH_2CH_2C\mathit{H_2}NHCO)_2$), 3.67 (AB-system, J = 13.1 Hz, 4H, 2*$C\mathit{H_2}$Ar), 7.15-7.35 (m, 12H, Ar, 2*NH). ^{13}C NMR (100 MHz, $CDCl_3$): 173.2, 139.6, 128.4, 128.0, 127.2, 67.9, 53.4, 38.7, 31.2, 29.7, 29.3, 29.1, 26.9, 19.5, 17.6. ESI-MS (m/z): calculated $C_{34}H_{54}N_4O_2$ [M+H$^+$] 551.4247, found 551.4232.

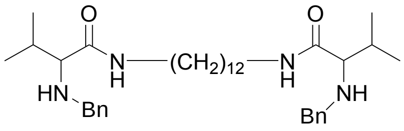

4e

4e: reaction solvent: CH_2Cl_2, yield 60%, yellow oil, R_f (hexane/EtOAc 1/1) 0.4. ^{1}H NMR (400 MHz, $CDCl_3$): 0.86 (d, J = 6.8 Hz, 6H, 2*CH_3), 0.94 (d, J = 6.8 Hz, 6H, 2*CH_3), 1.18-1.35 (m, 16H, $(C\mathit{H_2}C\mathit{H_2}C\mathit{H_2}C\mathit{H_2}CH_2CH_2NHCO)_2$), 1.44-1.56 (m, 4H, $(CH_2CH_2CH_2CH_2C\mathit{H_2}CH_2NHCO)_2$), 1.7 (br. s, 2H, 2*NH), 2.06-2.15 (m, 2H, 2*$C\mathit{H}(CH_3)_2$), 2.93 (d, J = 4.6 Hz, 2*C*H*), 3.20-3.30 (m, 4H, $(CH_2CH_2CH_2CH_2CH_2C\mathit{H_2}NHCO)_2$), 3.60 (AB-system, J = 13.1 Hz, 4H, 2*$C\mathit{H_2}$Ar), 7.20-7.40 (m, 12H, Ar, 2*NH). ^{13}C NMR (100 MHz, $CDCl_3$): 173.3, 139.7, 128.6, 128.1, 127.3, 68.0, 53.5, 38.8, 31.2, 29.8, 29.5, 29.3, 27.0, 19.6, 17.7. IR (cm^{-1}) 1630 (*CO*NH), 3300 (br. NH, CO*NH*). ESI-MS (m/z): calculated $C_{36}H_{58}N_4O_2$ [M+H$^+$] 579.4600, found 579.4642.

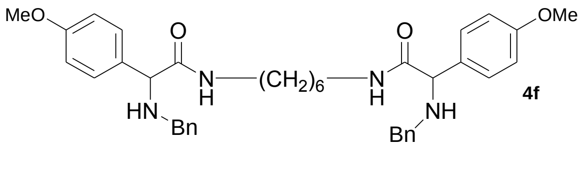

4f

4f: reaction solvent: MeOH, yield 31%, yellow oil, R_f (hexane/EtOAc 1/1) 0.4. ^{1}H NMR (400 MHz, $CDCl_3$): 1.22-1.29 (m, 4H, $(C\mathit{H_2}CH_2CH_2NHCO)_2$), 1.39-1.49 (m, 4H, $(CH_2C\mathit{H_2}CH_2NHCO)_2$), 1.9 (br. s, 2H, 2*NH), 3.14-3.26 (m, 4H,

($CH_2CH_2CH_2CH_2CH_2CH_2NHCO)_2$), 3.77 (s, 6H, 2*OC*H*$_3$), 3.70-3.80 (m, 4H, CH_2), 4.16 (s, 2H, 2*CH), 6.85 (d, *J* = 7.6 Hz, 4H, Ar), 7.03-7.10 (m, 2H, 2*NH), 7.24-7.36 (m, 14H, ArH). ^{13}C NMR (100 MHz, $CDCl_3$): 172.2, 159.4, 139.4, 131.5, 128.6, 128.4, 128.1, 127.3, 114.2, 66.3, 55.3, 52.4, 38.8, 29.5, 26.2. IR (cm^{-1}) 1630 (*CO*NH), 3270 (br. NH, CO*NH*). ESI-MS (m/z): calculated $C_{38}H_{46}N_4O_4$ [$M+H^+$] 623.3519, found 623.3528.

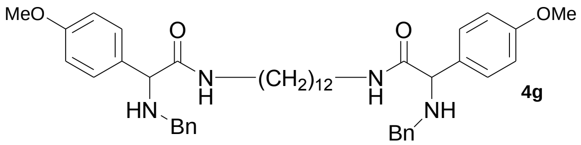

4g

4g:, reaction solvent: MeOH yield 33%, yellow oil, R_f (hexane/EtOAc 1/1) 0.4. ^1H NMR (400 MHz, $CDCl_3$): 1.18-1.30 (m, 16H, (C*H*$_2$C*H*$_2$C*H*$_2$C*H*$_2$$CH_2CH_2NHCO)_2$), 1.42-1.54 (m, 4H, ($CH_2CH_2CH_2CH_2$C*H*$_2$$CH_2NHCO)_2$), 1.9 (br. s, 2H, 2*NH), 3.15-3.27 (m, 4H, ($CH_2CH_2CH_2CH_2CH_2$C*H*$_2$NHCO)$_2$), 3.77 (s, 6H, 2*OC*H*$_3$), 3.70-3.80 (m, 4H, CH_2), 4.16 (s, 2H, 2*CH), 6.85 (d, *J* = 8.6 Hz, 4H, Ar), 7.05-7.10 (m, 2H, 2*NH), 7.24-7.36 (m, 14H, ArH). ^{13}C NMR (100 MHz, $CDCl_3$): 172.1, 159.4, 139.4, 131.5, 128.5, 128.4, 128.1, 127.3, 114.2, 66.3, 55.2, 52.4, 39.1, 29.6, 29.5, 29.2, 26.7. IR (cm^{-1}) 1650 (*CO*NH), 3250 (br. NH, CO*NH*). ESI-MS (m/z): calculared $C_{44}H_{58}N_4O_4$ [$M+H^+$] 706.4458, found 706.4411.

2.3. *Cleavage of the benzyl group*

A solution of $HCOONH_4$ (1 g in 5 ml H_2O) was added to a solution of the corresponding diamide 4 (1 mmol) in 10 mL of MeOH. The catalyst, Pd/C, (100 mg, 5%) was added and the mixture heated under reflux for 5 h. The mixture was concentrated and treated with a solution of K_2CO_3 in water. The product was extracted with CH_2Cl_2 (3x30 ml), the organic layer dried (K_2CO_3) and concentrated in vacuo. The residue was purified by column chromatography (CH_2Cl_2 : MeOH 10:1). The resulting product (colorless oil or white solid) can be converted into corresponding hydrochloride by treatment with gaseous HCl in MeOH.

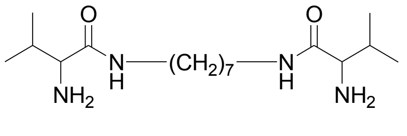

5b

5b: Yield 85%, colorless oil, R_f ($CH_3CN/EtOH/NH_3$ 80/12/8) 0.6. ^1H NMR (400 MHz, $CDCl_3$): 0.76 (d, *J* = 7.1 Hz, 6H, 2*CH_3), 0.92 (d, *J* = 7.1 Hz, 6H, 2*CH_3), 1.20-1.34 (m, 6H, C*H*$_2$(C*H*$_2$$CH_2CH_2NHCO)_2$), 1.39-1.50 (m, 4H, CH_2(CH_2C*H*$_2$$CH_2NHCO)_2$), 2.19-2.30 (m, 2H,

$2{*}C\mathit{H}(CH_3)_2$), 3.11-3.26 (m, 6H, $2{*}C\mathit{H}$, $CH_2(CH_2CH_2C\mathit{H}_2NHCO)_2$), 7.3 (br. s, 2H, 2*NH). ^{13}C NMR (100 MHz, $CDCl_3$): 174.2, 60.1, 38.8, 30.7, 29.5, 28.7, 26.7, 19.6, 15.9. IR (cm^{-1}) 1644 (*CO*NH), 3290 (br., NH, NH_2). ESI-MS (m/z): calculated $C_{17}H_{36}N_4O_2$ [M+H$^+$] 328.2838, found 328.2814.

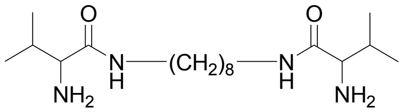

5c

5c: Yield 90%, colorless oil, R_f ($CH_3CN/EtOH/NH_3$ 80/12/8) 0.6. ^1H NMR (400 MHz, $CDCl_3$): 0.78 (d, J = 6.8 Hz, 6H, $2{*}CH_3$), 0.96 (d, J = 6.8 Hz, 6H, $2{*}CH_3$), 1.20-1.34 (m, 12H, $2{*}NH_2$, $(C\mathit{H}_2C\mathit{H}_2CH_2CH_2NHCO)_2$), 1.40-1.50 (m, 4H, $(CH_2CH_2C\mathit{H}_2CH_2NHCO)_2$), 2.20-2.32 (m, 2H, $2{*}C\mathit{H}(CH_3)_2$), 3.10-3.30 (m, 6H, $2{*}C\mathit{H}$, $(CH_2CH_2CH_2C\mathit{H}_2NHCO)_2$), 7.3 (br. s, 2H, 2*NH). ^{13}C NMR (100 MHz, $CDCl_3$): 174.2, 60.1, 38.8, 30.7, 29.6, 29.0, 26.8, 19.7, 15.9. IR (cm^{-1}) 1640 (*CO*NH), 3290 (br., NH, NH_2). ESI-MS (m/z): calculated $C_{18}H_{38}N_4O_2$ [M+H$^+$] 343.2995, found 343.3068.

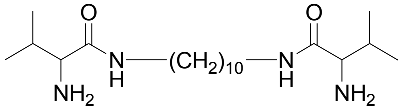

5d

5d: yield 79%, colorless oil, R_f ($CH_3CN/EtOH/NH_3$ 80/12/8) 0.6. ^1H NMR (400 MHz, $CDCl_3$): 0.80 (d, J = 6.8 Hz, 6H, $2{*}CH_3$), 0.96 (d, J = 6.8 Hz, 6H, $2{*}CH_3$), 1.20-1.34 (m, 16H, $2{*}NH_2$, $(C\mathit{H}_2C\mathit{H}_2C\mathit{H}_2CH_2CH_2NHCO)_2$), 1.40-1.50 (m, 4H, $(CH_2CH_2CH_2C\mathit{H}_2CH_2NHCO)_2$), 2.20-2.32 (m, 2H, $2{*}C\mathit{H}(CH_3)_2$), 3.10-3.30 (m, 6H, $2{*}C\mathit{H}$, $(CH_2CH_2CH_2CH_2C\mathit{H}_2NHCO)_2$). ^{13}C NMR (100 MHz, $CDCl_3$): 174.4, 60.2, 38.9, 30.8, 29.7, 29.4, 29.2, 26.9, 19.7, 15.9. IR (cm^{-1}) 1639 (*CO*NH), 3290 (br., NH, NH_2). ESI-MS (m/z): calculated $C_{20}H_{42}N_4O_2$ [M+H$^+$] 370.3308, found 370.3279.

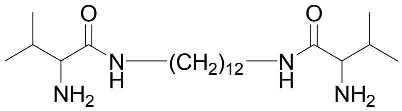

5e

5e: yield 65%, mp 95-97ºC, R_f ($CH_3CN/EtOH/NH_3$ 80/12/8) 0.6. White solid (hydrochloride) ^1H NMR (400 MHz, DMSO): 0.81 (d, J = 6.8 Hz, 6H, $2{*}CH_3$), 0.86 (d, J = 6.8 Hz, 6H, $2{*}CH_3$), 1.15-1.28 (m, 16H, $(C\mathit{H}_2C\mathit{H}_2C\mathit{H}_2C\mathit{H}_2CH_2CH_2NHCO)_2$), 1.33-1.42 (m, 4H, $(CH_2CH_2CH_2CH_2C\mathit{H}_2CH_2NHCO)_2$), 1.84-1.94 (m, 2H, $2{*}C\mathit{H}(CH_3)_2$), 2.93-3.05 (m, 2H, $2{*}C\mathit{H}$),

3.06-3.13 (m, 4H, ($CH_2CH_2CH_2CH_2CH_2C\textit{H}_2$NHCO)$_2$), 8.00-8.07 (m, 2H, 2*NH). ^{13}C NMR (100 MHz, DMSO): 171.9, 59.2, 38.3, 31.0, 29.1, 29.0, 28.9, 28.7, 26.4, 19.1, 17.4. IR (cm^{-1}) 1638 (*CO*NH), 3290 (br., NH, NH_2). ESI-MS (m/z): calculated $C_{22}H_{46}N_4O_2$ [M$^+$] 398.3620, found 399.3694.

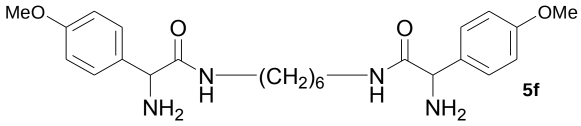

5f

5f: yield 60%, yellow oil, R$_f$ (CH_3CN/EtOH/NH_3 80/12/8) 0.6. ^{1}H NMR (400 MHz, DMSO-*d*$_6$): 1.10-1.40 (m, 12H, 2*NH_2, ($C\textit{H}_2C\textit{H}_2CH_2$NHCO)$_2$), 2.94-3.06 (m, 4H, ($CH_2CH_2C\textit{H}_2$NHCO)$_2$), 3.70 (s, 6H, 2*OC*H*$_3$), 4.25 (s, 2H, 2*CH), 6.84 (d, *J* = 8.6 Hz, 4H, Ar), 7.27 (d, *J* 8.6 Hz, 4H, Ar), 7.94-7.98 (m, 2H, 2*NH). ^{13}C NMR (100 MHz, DMSO-*d*$_6$): 173.1, 158.3, 134.9, 127.7, 113.4, 58.3, 55.0, 38.3, 29.0, 25.9. IR (cm^{-1}) 1645 (*CO*NH), 3290 (br., NH, NH_2). ESI-MS (m/z): calculated $C_{24}H_{34}N_4O_4$ [M+H$^+$] 443.2580, found 443.2653.

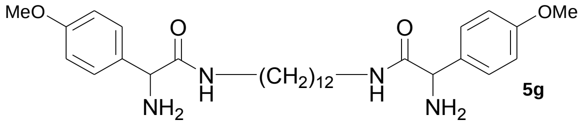

5g

5g: yield 66%, R$_f$ (CH_3CN/EtOH/NH_3 80/12/8) 0.6. White solid (hydrochloride): ^{1}H NMR (400 MHz, DMSO-*d*$_6$): 1.10-1.25 (m, 16H, ($C\textit{H}_2C\textit{H}_2C\textit{H}_2C\textit{H}_2CH_2CH_2$NHCO)$_2$), 1.30-1.40 (m, 4H, ($CH_2CH_2CH_2CH_2C\textit{H}_2CH_2$NHCO)$_2$), 3.90-3.05 (m, 4H, ($CH_2CH_2CH_2CH_2CH_2C\textit{H}_2$NHCO)$_2$), 3.70 (s, 6H, 2*OC*H*$_3$), 4.30 (s, 2H, 2*CH), 6.85 (d, *J* = 8.6 Hz, 4H, Ar), 7.28 (d, *J* = 8.6 Hz, 4H, Ar), 7.99-8.03 (m, 2H, 2*NH). ^{13}C NMR (100 MHz, DMSO-*d*$_6$): 172.6, 158.4, 134.1, 127.9, 113.4, 57.9, 55.0, 39.1, 38.4, 29.1, 29.0, 28.8, 26.3. IR (cm^{-1}) 1639 (*CO*NH), 3290 (br., NH, NH_2). ESI-MS (m/z): calculated $C_{30}H_{46}N_4O_4$ [M+H$^+$] 526.3519, found 526.3449.

3. Investigation of Biological Activity

3.1. *RNA isolation*

Total RNA were isolated from 10% brain suspensions from ICR mice infected with the tick-borne encephalitis virus (TBEV) (strain 2530 of Siberian genetic subtype) by lysis in guanidine isothiocyanate, phenol-chloroform de-proteinization and subsequent alcohol precipitation.[1]

3.2. *Cleavage of nucleic acids*

Artificial ribonucleases were dissolved in RNase-free DEPC-treated water in concentrations from $5x10^{-7}$ M to $5x10^{-3}$ M. Total RNA (5-10 µg in 5 µl) was incubated with 5 µl RNase-mimetic solution at 37°C for 2 h. Double stranded DNA cleavage experiments were performed using pBR322-TBEVS* recombinant plasmid with a cloned full-length DNA copy of the TBEV (Sofjin strain)[2] and ssDNA cleavage experiments were performed using total cDNA in RNase-free water. Incubation of ds or ssDNA was performed at 37 °C in the presence and absence of the test compounds for 2 h. Then reaction products were assayed in a reverse transcription reaction with subsequent real-time PCR with a TaqMan probe.

3.3. *Quantitation of the TBEV RNA, cDNA and plasmid DNA by reverse transcription with subsequent real-time PCR*

Reverse transcription was performed by using “Reverta-L” kit (“AmpliSense”, Moscow, Russia) according to manufacturer’s instruction. Real-time PCR was carried out using the following primers and probes specific to NS1[3] with minor modifications, TaqF DNA polymerase (“AmpliSense”, Moscow, Russia) and iCycler 4 and 5 (BioRad, USA).

NS1: NS1F – (5’-YTTCAGACAGGAACCAACAC-3’),

NS1R - (5’-CAGTTYCTCARGTCAGTGAC-3’),

NS1-probe–(5’-FAM-CATGGCGGTCCACACTGACCAAA-BHQ1-3’).

---

1. Chomczynski, P.; Sacchi, N. *Anal Biochem.* **1987**, *162*, 156-159.

2. Dobrikova, E. Yu.; Pletnev, A. G. *Bioorg Khim.* **1995**, *21*, 528-534.

3. Bakhvalova, V. N.; Potapova, O. F.; Panov, V.V.; Morozova, O.V. *Virus Research* **2009**, *140*, 172–178.

The primers and hydrolysis probe specific to the TBEV NS1 gene were synthesized in the Novosibirsk Institute of Chemical Biology and Fundamental Medicine of the Siberian Branch of the Russian Academy of Sciences.

TBEV is a human pathogenic member of the *Flavivirudae* family of RNA-containing viruses of positive polarity. The genome of tick-borne flaviviruses consists of a single-stranded RNA molecule of approximately 11,000 nucleotides in length and positive polarity.[4] Secondary structures of 5'- and 3'-terminal untranslated regions of genomes of the flaviviruses as well as encapsidation of the genomic RNA with structural proteins of virions protect the virus genomes from possible cellular or extracellular RNases. Therefore, for estimation of nuclease activity of novel small peptidomimetics, cleavage of the flavivirus RNA near the middle of linear single-stranded RNA in the region of NS1 gene was studied by means of RT-qPCR with primers and fluorescent probe specific to the TBEV NS1 gene. Cleavage near the middle of the viral genomic RNA is believed to result in a decrease of its virulence. According to previous estimations[5], the lengths of products of RNA cleavage with artificial RNases were less than 100 nucleotides so the TBEV NS1 specific PCR fragment length of 156 bp seemed to be convenient.

In order to optimize RNA cleavage *in vitro*, approximately 2 μg of total RNA isolated from laboratory mouse brain infected with the TBEV were incubated with 2.5 mM peptidomimetics at 37°C for 2 hours. Denaturating electrophoresis in SDS-agarose gel revealed complete cleavage of total RNA (Figure S1) and RT- real time PCR showed complete destruction of the TBEV RNA (Figure S2).

4. Pletnev, A. G.; Yamshchikov, V. F.; Blinov, V. M. *Virology* **1990**, *174*, 250-263.

5. Koroleva, L. S.; Serpokrylova, I. Yu.; Vlassov, V.V.; Silnikov, V. N. *Protein Peptide. Lett.*, **2007**, *14*, 151-163.

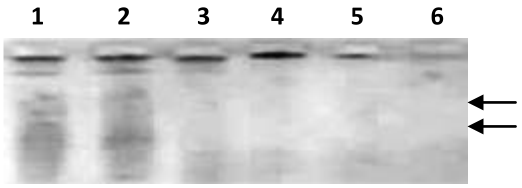

Lane 1 corresponds to original 2 μg RNA stored at -20^{0}C. Lane 2 – total RNA after incubation in water at +37^{0}C for 2 h, Lane 3-4 – total RNA after incubation with 2.5 mM **5e** solution after storage at +4^{0}C for 2 months (lane 3) or diluted immediately before experiment (lane 4). Lane 5-6 – total RNA after incubation with 2.5 mM **5g** solution after storage at +4^{0}C for 2 months (lane 5) or diluted immediately before experiment (lane 6). Right arrows show positions of 28S and 18S rRNA from mouse brain cells.

Figure S1. Results of electrophoresis in 1.5% agarose gel with 1% SDS of total RNA cleavage products.

The concentration of compounds **5e** and **5g**, optimal for RNA cleavage, was determined by varying the concentration from 2.5x10^{-3} to 2.5x10^{-7} M (Fig. S2 A and B). Dependence of optimal RNA cleavage on concentrations of peptidomimetics was not evident. For both **5e (A)** with an aliphatic substituent and **5g** (B) with an aromatic one, complete cleavage of 2 μg RNA was observed at concentration 2.5 mM.

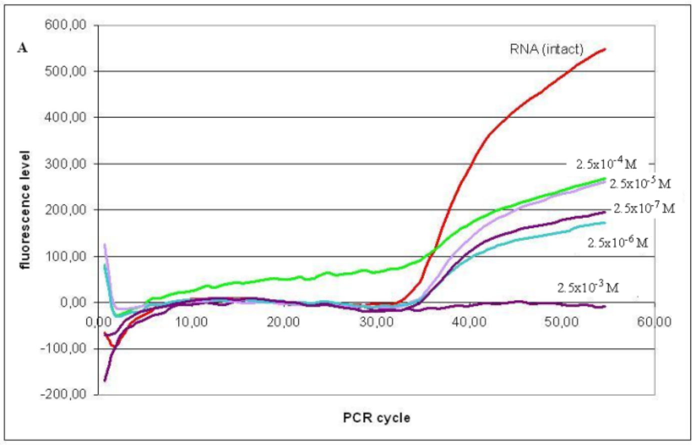

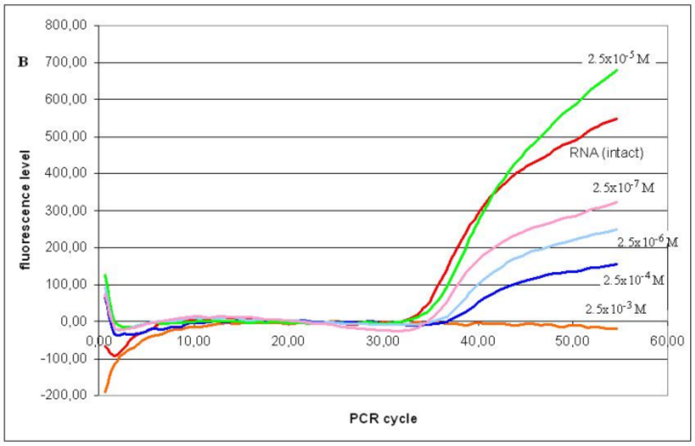

Figure S2. Results of RT-qPCR of the TBEV RNA cleavage products in presence of **5e** (A) or **5g** (B) in concentration from $2.5x10^{-3}$ to $2.5x10^{-7}$ M at 37^oC for 2 hours in H_2O.

To analyze DNase activity of the novel compounds both double stranded circular recombinant plasmid DNA with a cloned full-length TBEV copy of genome and single stranded cDNA after reverse transcription of the TBEV RNA from the infected mouse brain with a random N_6 primer was used. No destruction of single-stranded cDNA was observed after incubation with 2.5 mM solutions of compounds **5e** or **5g** for 2 h at 37^0C (Figure S3). However, these compounds could partly cleave double-stranded plasmid DNA (Figure S4).

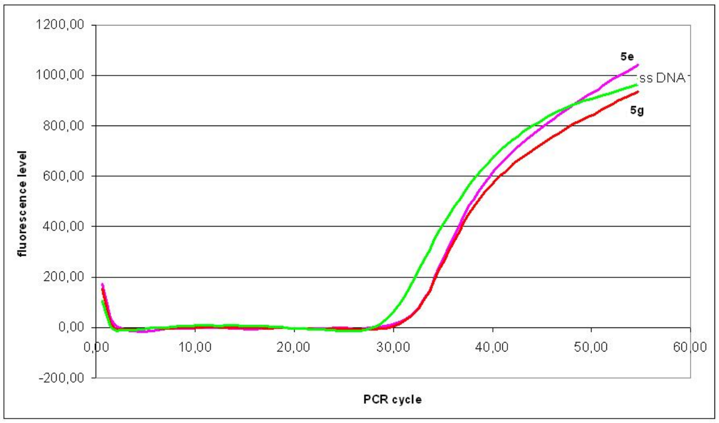

Figure S3. Results of RT-qPCR of the TBEV single-stranded cDNA cleavage products in presence of peptidomimetics **5e** and **5g** (2.5 mM) at 37^oC for 2 hours in H_2O.

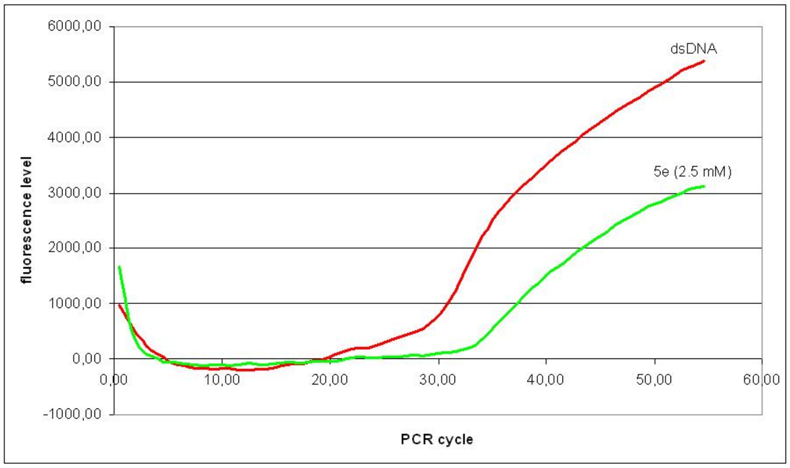

Figure S4. Results of RT-qPCR of double-stranded plasmid DNA cleavage products in the presence of peptidomimetics **5e** at 37°C for 2 hours in H_2O.

The novel peptidomimetics were shown not to inhibit reverse transcription and PCR as shown by PCR with the TBEV cDNA in the presence or absence of the new compounds and described in details elsewhere. Since some diamines are known to cleave RNA, therefore, at beginning control incubation of the TBEV RNA in the presence of 1,12-diaminododecane (2.5 mM and 25 mM) was performed for 2 hours at 37°C in H_2O but no cleavage was observed (Figure S5).

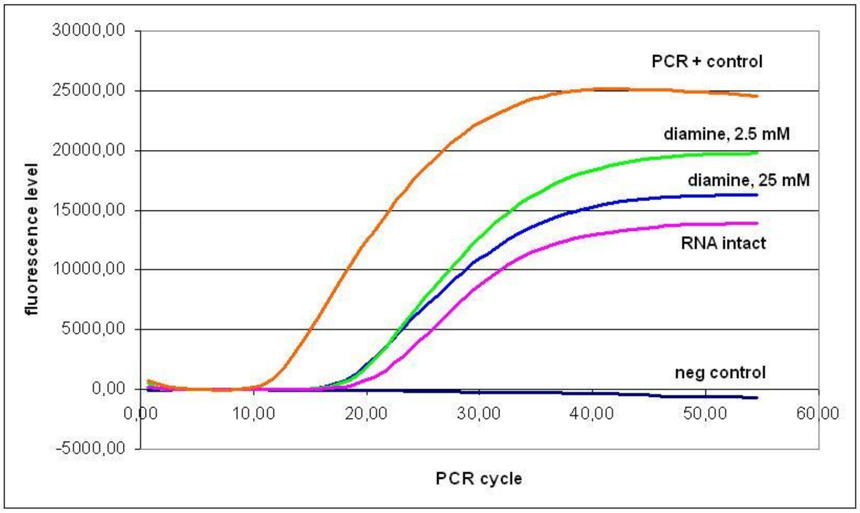

Figure S5. Results of real time PCR of the TBEV RNA cleavage products in presence of 1,12-diaminododecane at 37°C for 2 hours in H_2O.

4. Selected NMR Spectra

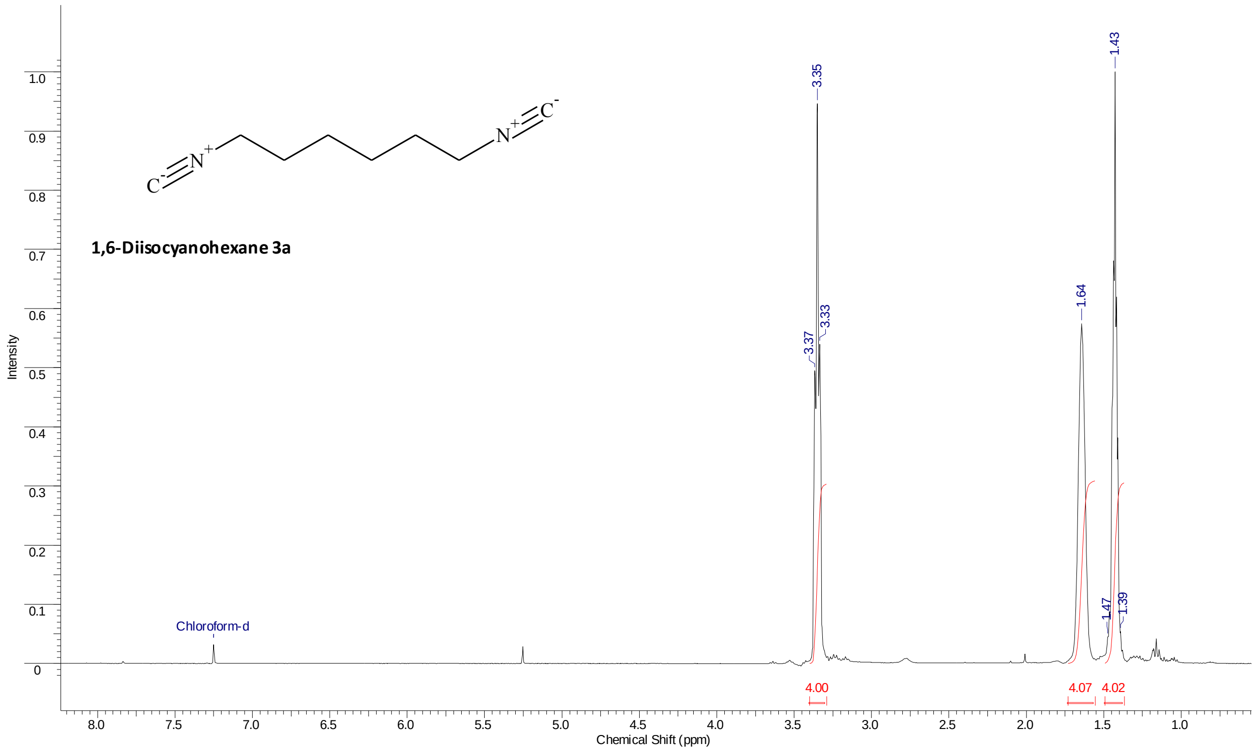

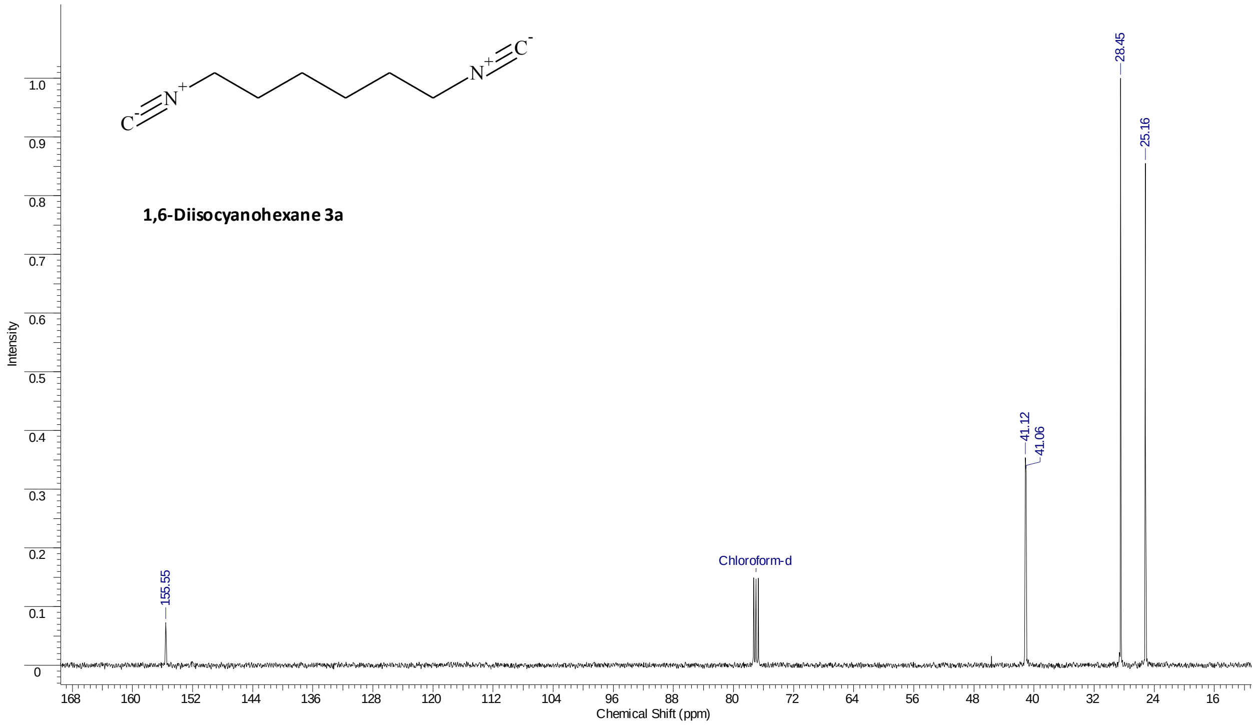

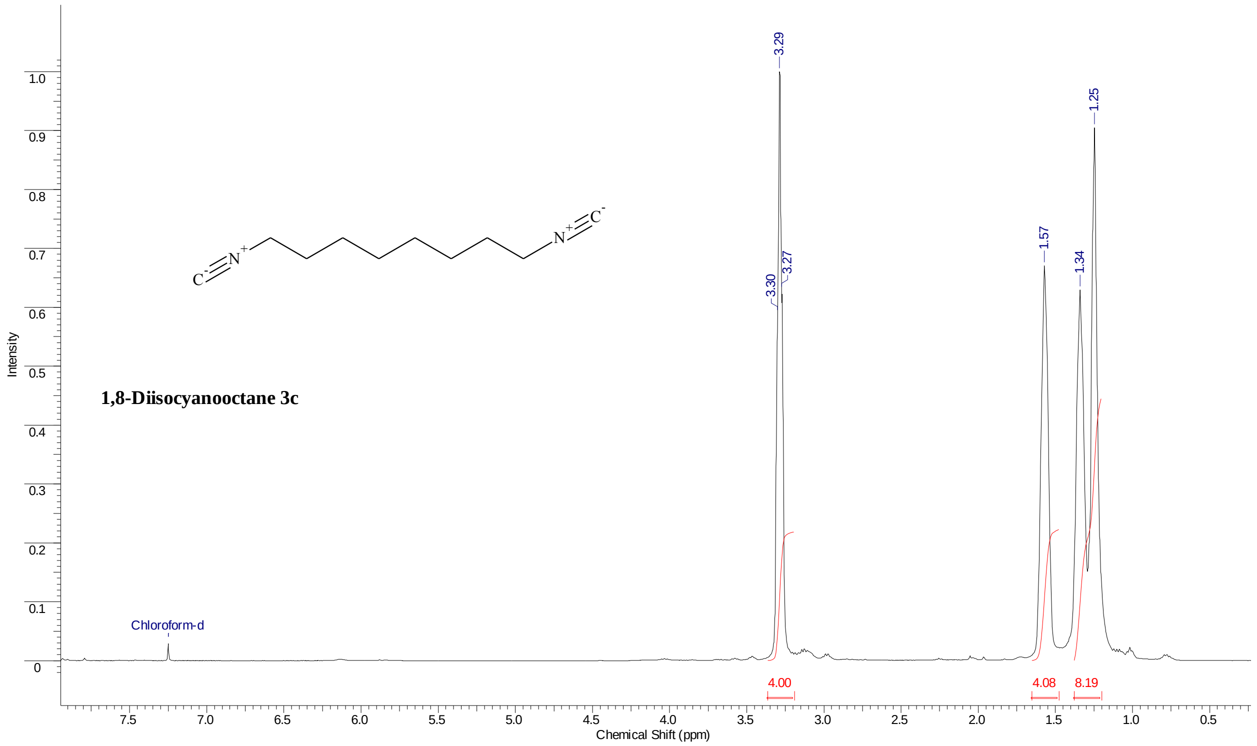
1,8-Diisocyanooctane 3c
Chloroform-d
3.29
3.30
3.27
1.57
1.34
1.25
4.00
4.08
8.19
Intensity
Chemical Shift (ppm)

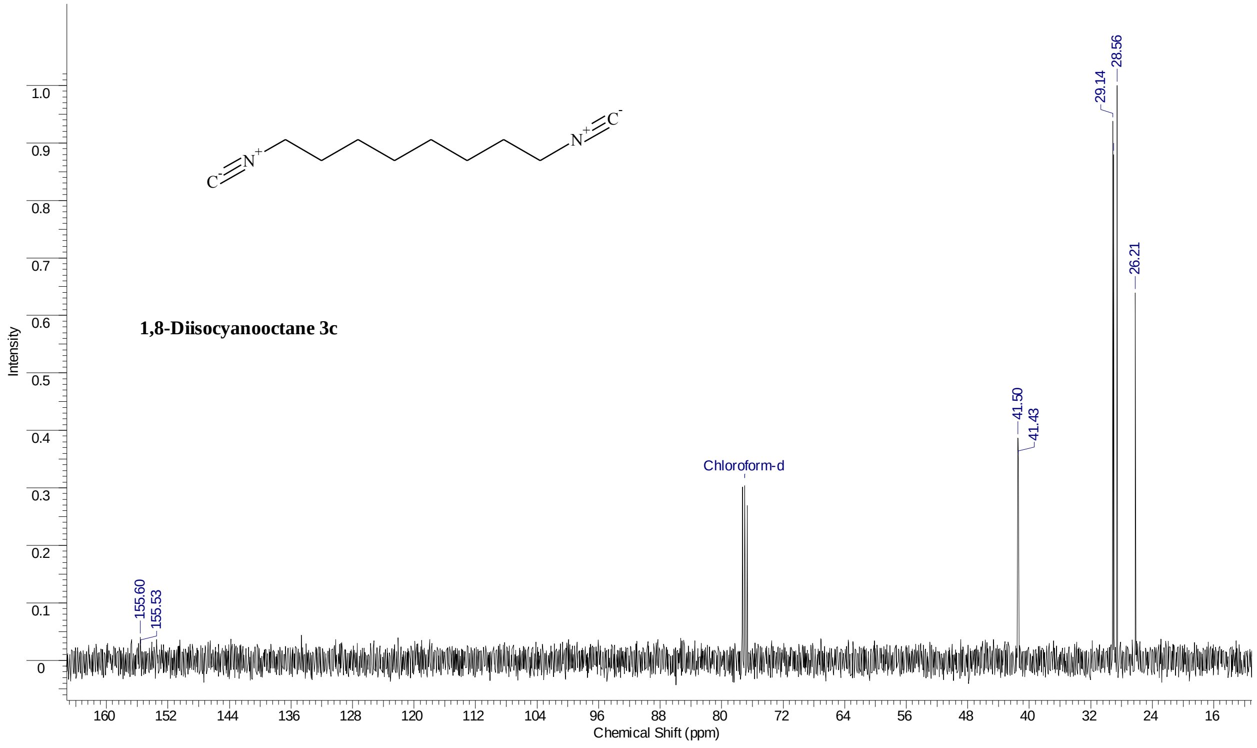
1,8-Diisocyanooctane 3c
Chloroform-d
155.60
155.53
41.50
41.43
29.14
28.56
26.21
Intensity
Chemical Shift (ppm)

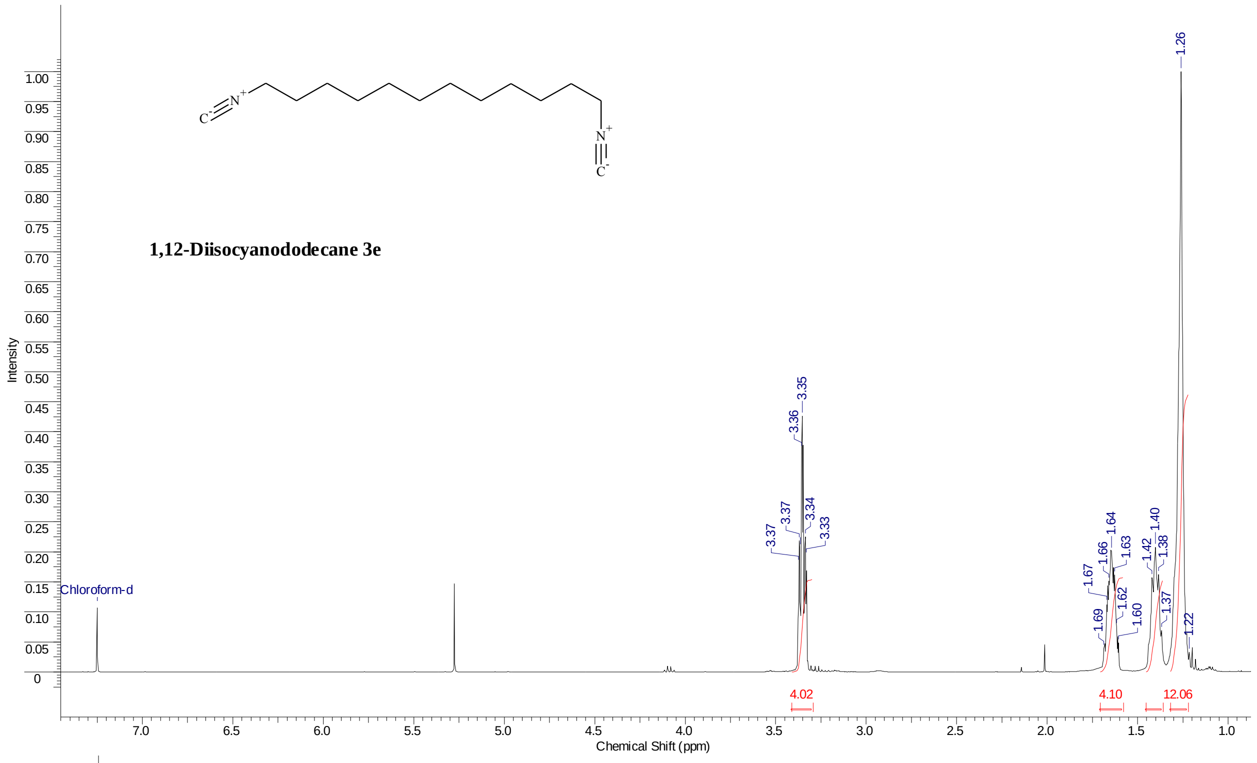
1,12-Diisocyanododecane 3e
Chloroform-d
Intensity
Chemical Shift (ppm)
1.26
3.35
3.36
3.37
3.37
3.34
3.33
1.67
1.66
1.64
1.63
1.69
1.62
1.60
1.42
1.40
1.38
1.37
1.22
4.02
4.10
12.06

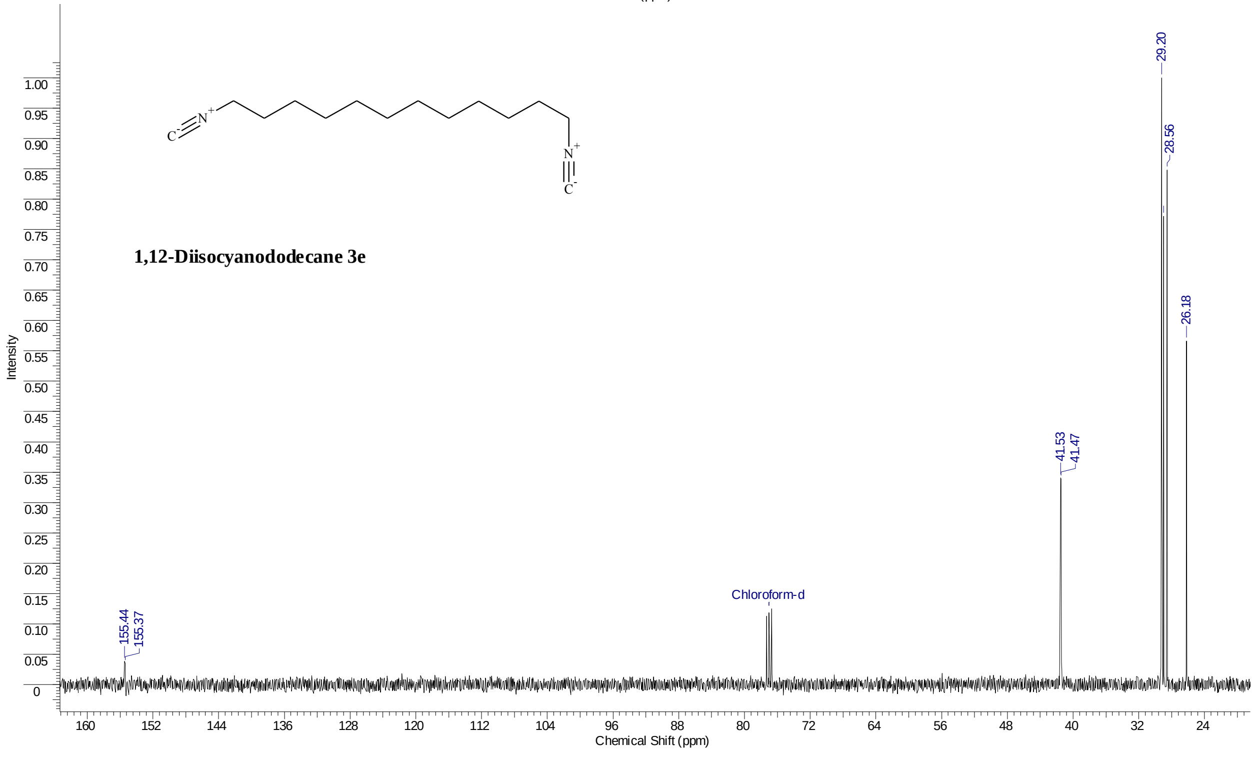
1,12-Diisocyanododecane 3e
Chloroform-d
Intensity
Chemical Shift (ppm)
29.20
28.56
26.18
41.53
41.47
155.44
155.37

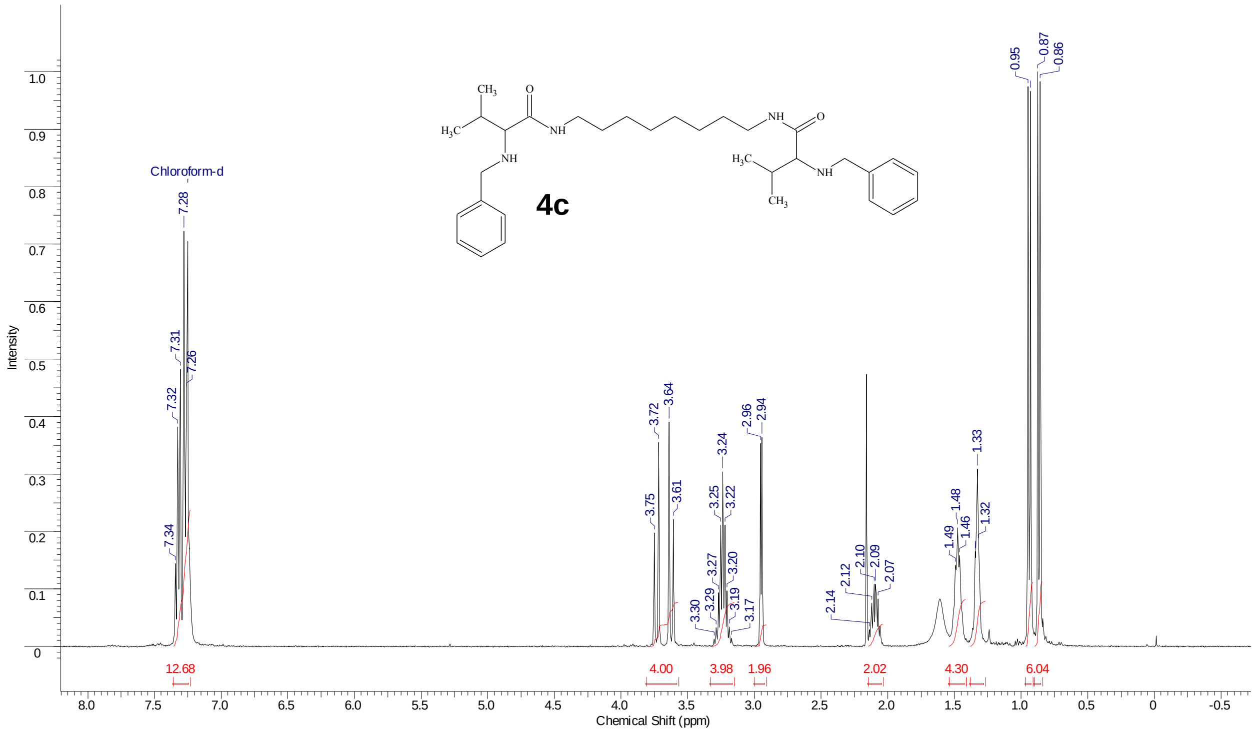
4c
Chloroform-d
Intensity
Chemical Shift (ppm)
12.68
4.00
3.98
1.96
2.02
4.30
6.04

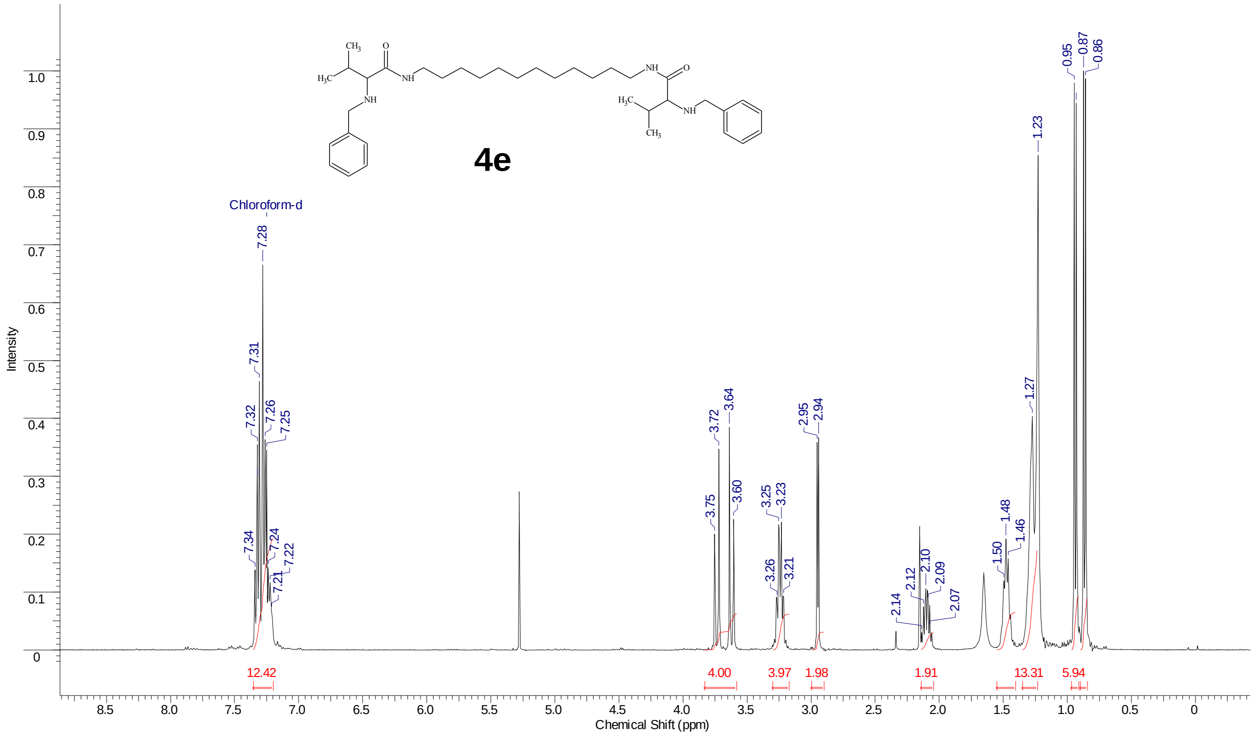
4e
Chloroform-d
Intensity
Chemical Shift (ppm)
12.42
4.00
3.97
1.98
1.91
13.31
5.94

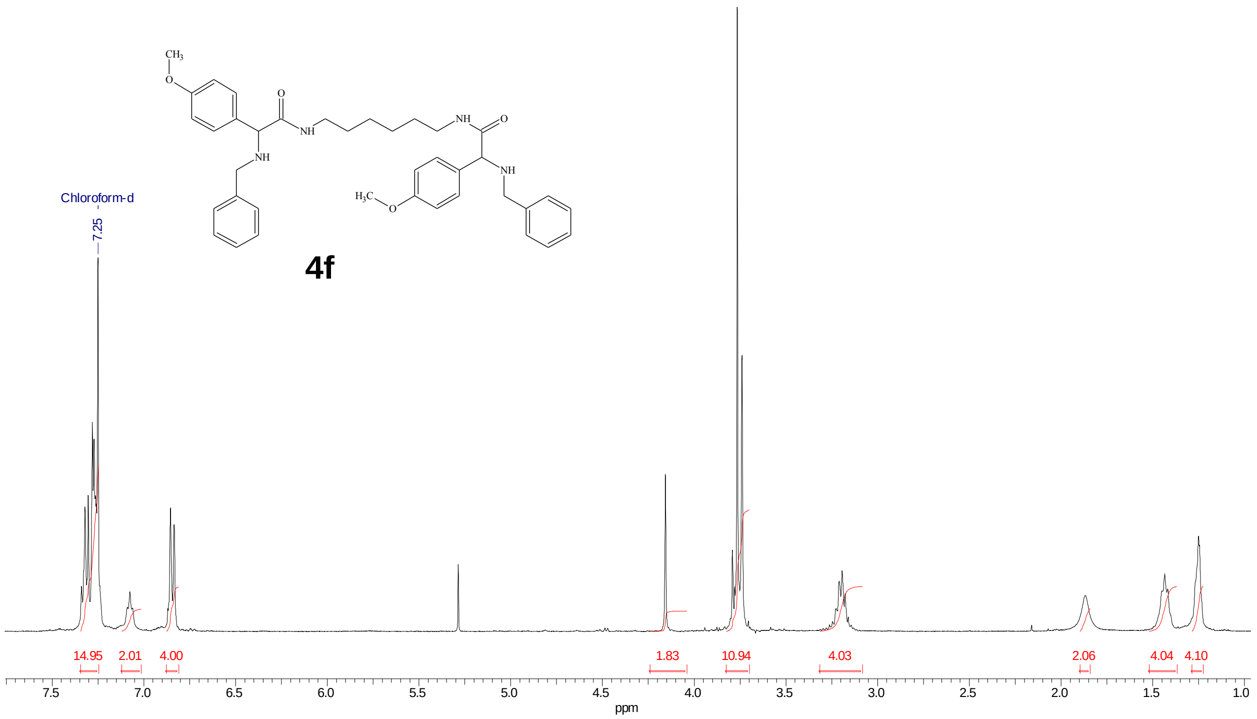
4f
Chloroform-d
7.25
14.95
2.01
4.00
1.83
10.94
4.03
2.06
4.04
4.10
ppm

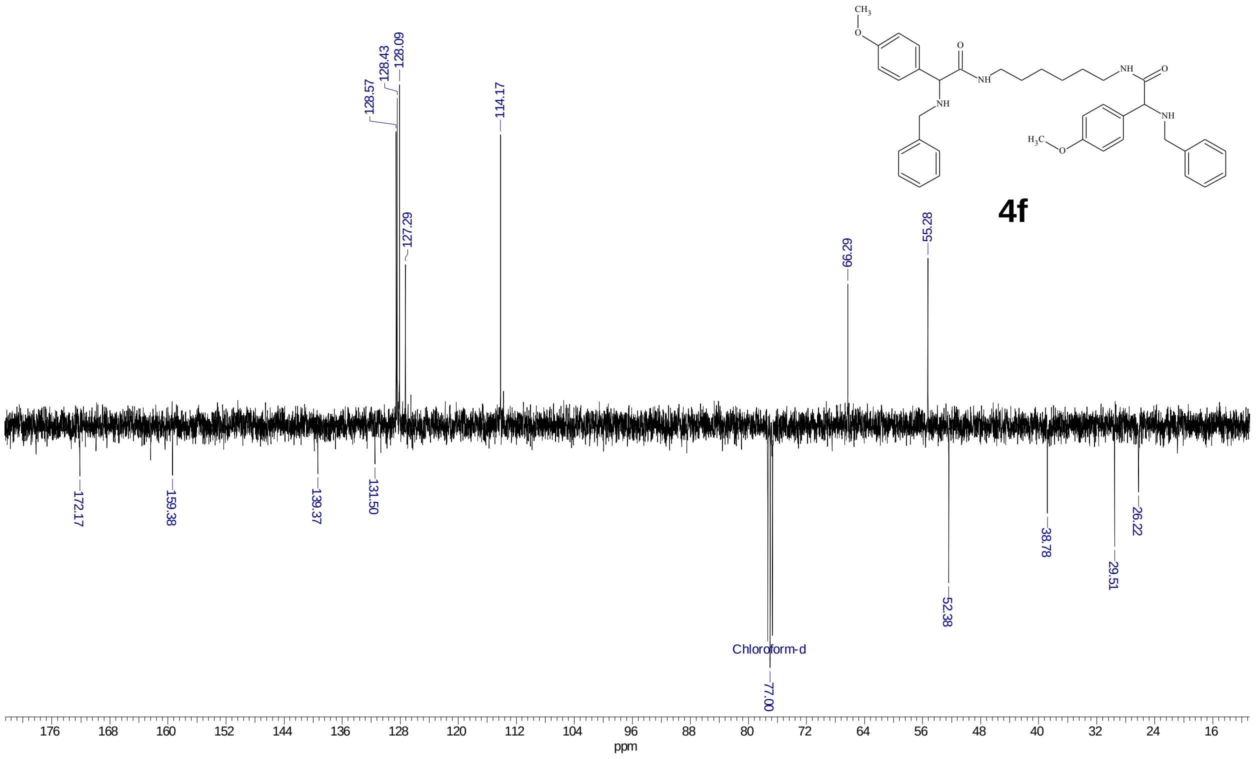
4f
128.57
128.43
128.09
114.17
127.29
66.29
55.28
172.17
159.38
139.37
131.50
38.78
29.51
26.22
52.38
Chloroform-d
77.00
ppm

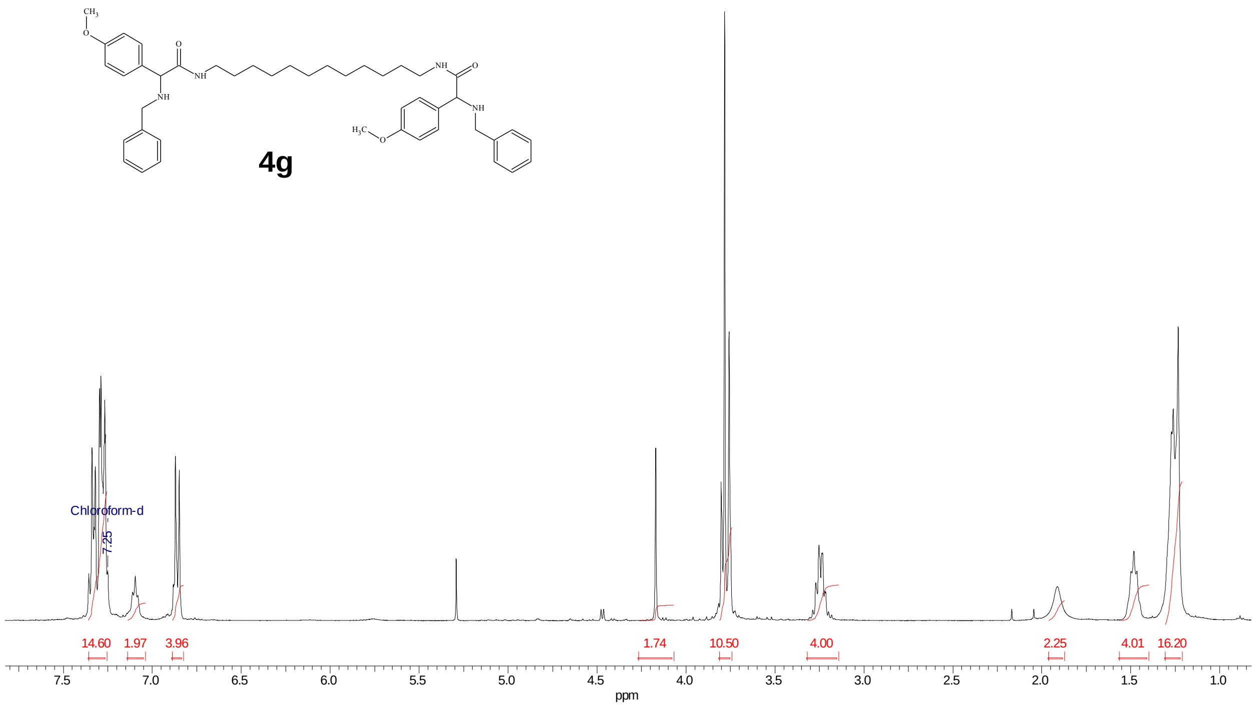
4g
Chloroform-d
7.25
14.60 1.97 3.96 1.74 10.50 4.00 2.25 4.01 16.20
7.5 7.0 6.5 6.0 5.5 5.0 4.5 4.0 3.5 3.0 2.5 2.0 1.5 1.0
ppm

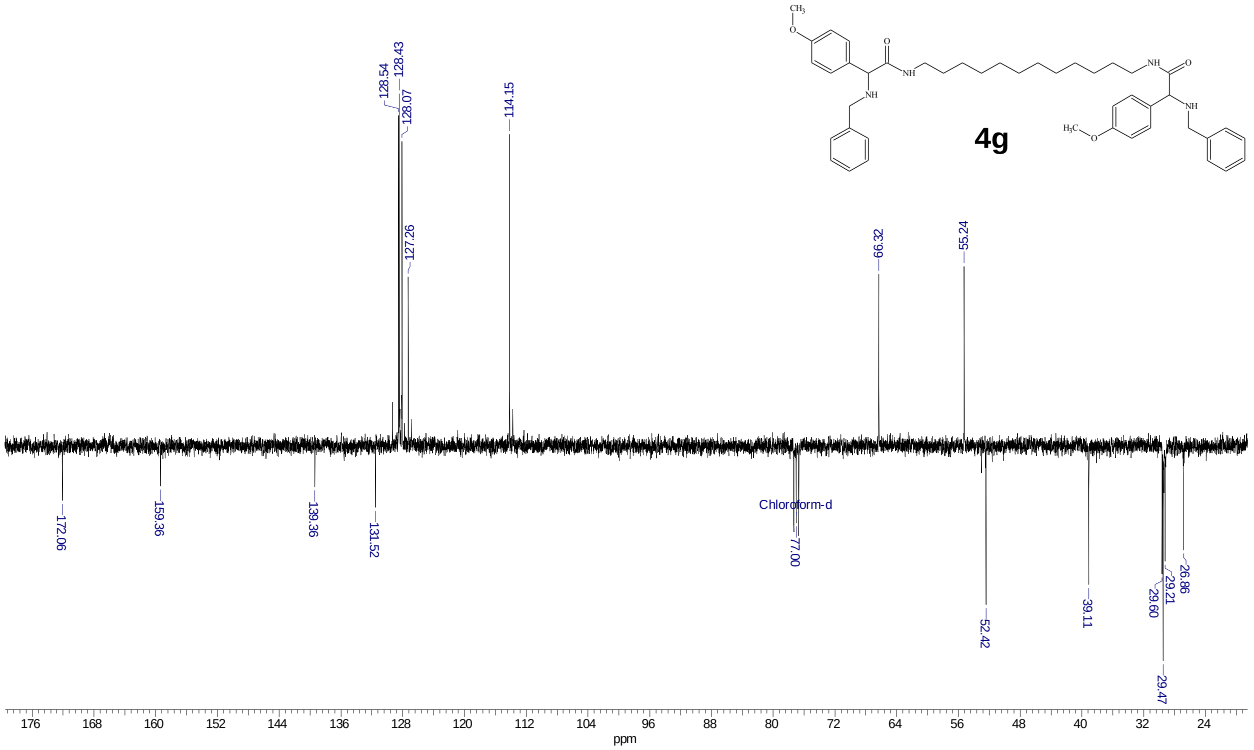
4g
128.54 128.43 128.07 127.26 114.15 66.32 55.24
172.06 159.36 139.36 131.52
Chloroform-d
77.00 52.42 39.11 29.60 29.21 26.86 29.47
176 168 160 152 144 136 128 120 112 104 96 88 80 72 64 56 48 40 32 24
ppm

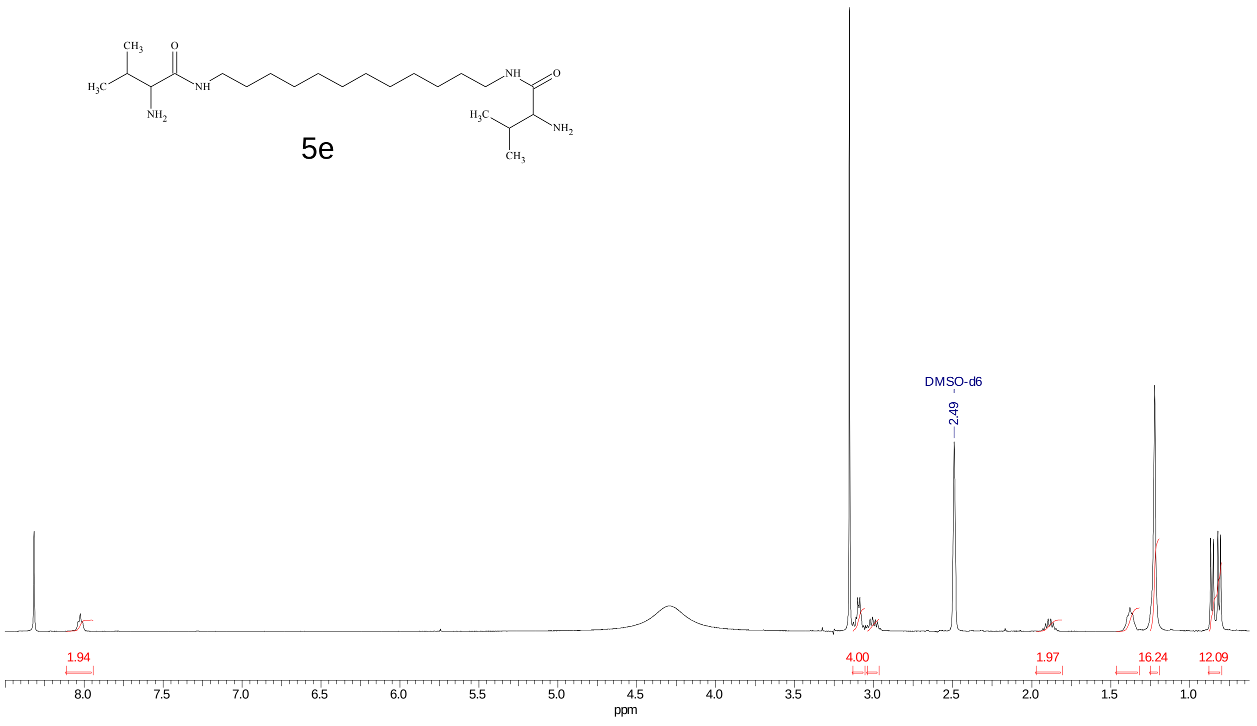
5e
DMSO-d6
2.49
1.94
4.00
1.97
16.24
12.09
ppm

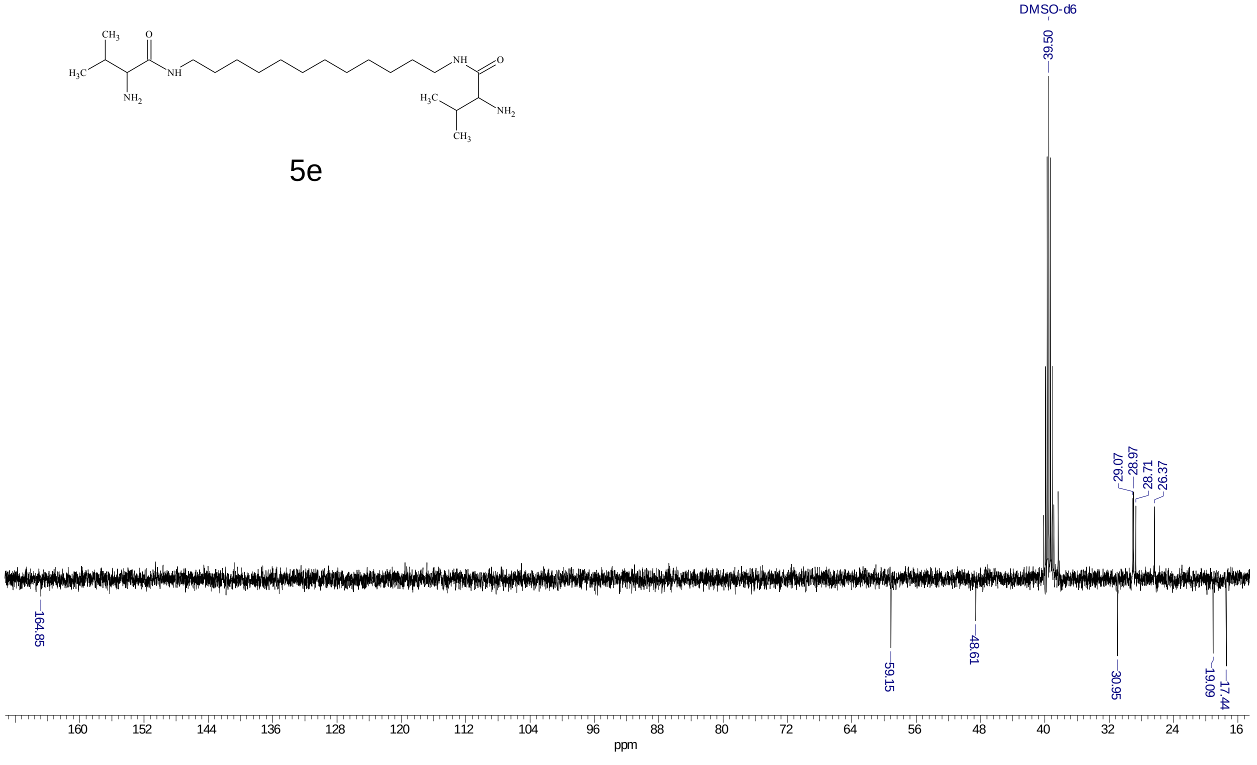
5e
DMSO-d6
39.50
29.07
28.97
28.71
26.37
164.85
59.15
48.61
30.95
19.09
17.44
ppm

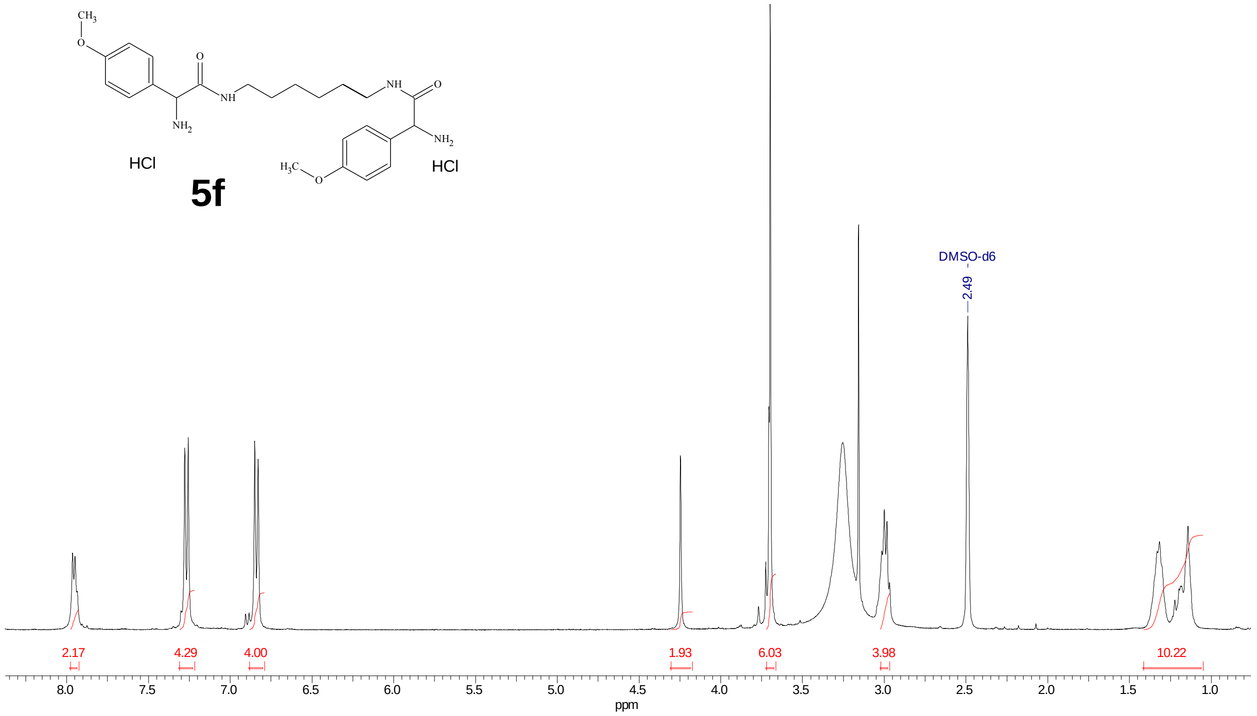
HCl
5f
HCl
DMSO-d6
2.49
2.17
4.29
4.00
1.93
6.03
3.98
10.22
ppm

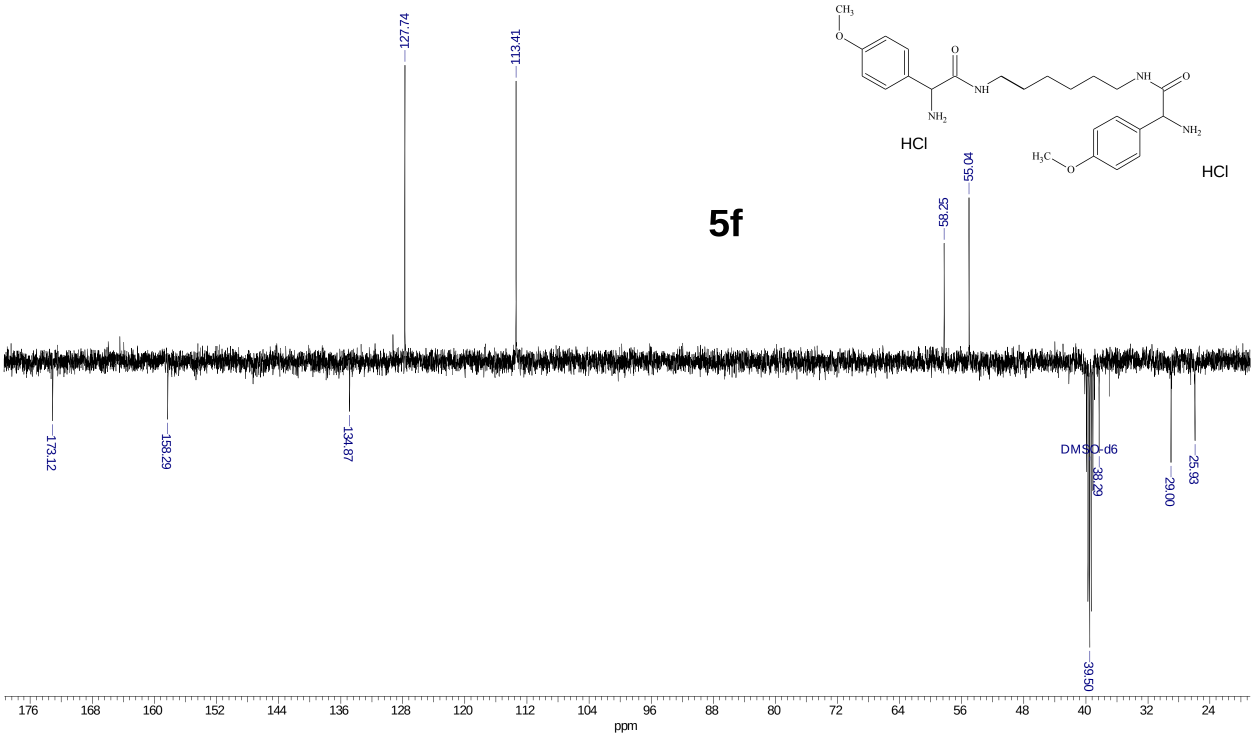
127.74
113.41
HCl
HCl
5f
55.04
58.25
173.12
158.29
134.87
DMSO-d6
38.29
29.00
25.93
39.50
ppm

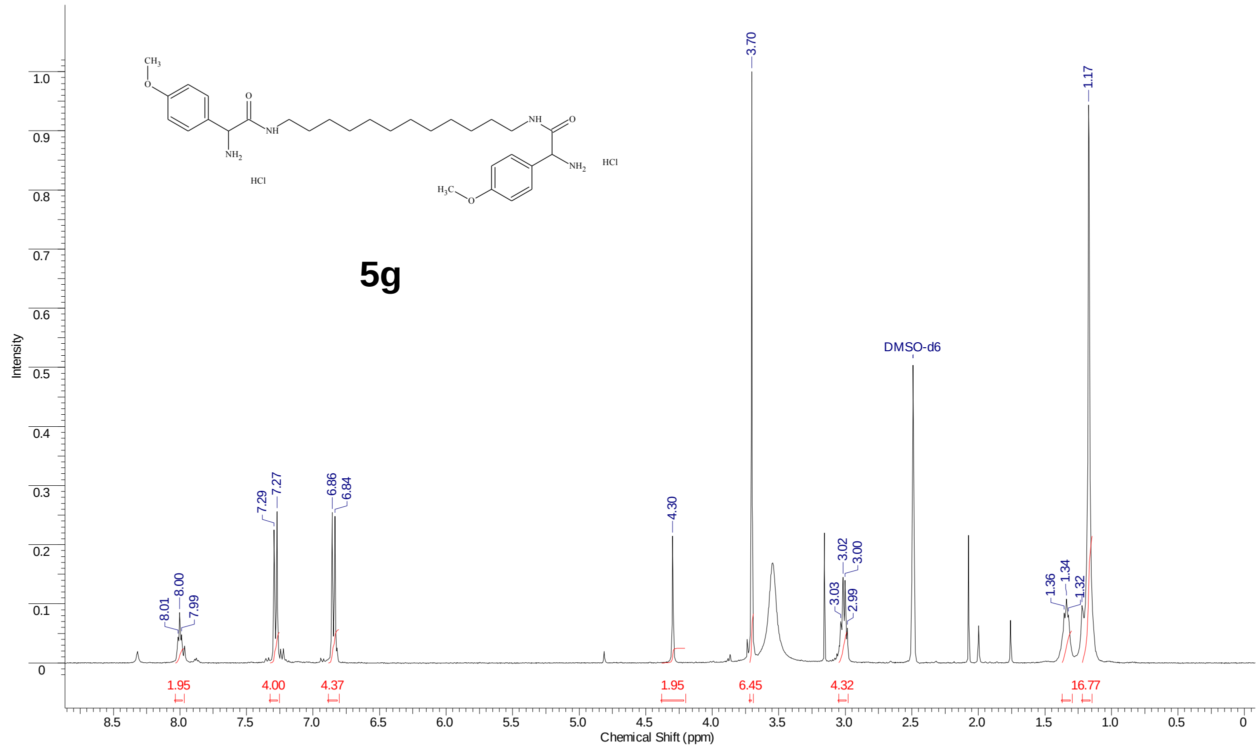
5g
CH3
O
NH
NH2
HCl
H3C
DMSO-d6
3.70
1.17
7.29
7.27
6.86
6.84
4.30
8.01
8.00
7.99
3.03
3.02
3.00
2.99
1.36
1.34
1.32
1.95
4.00
4.37
1.95
6.45
4.32
16.77
Intensity
Chemical Shift (ppm)

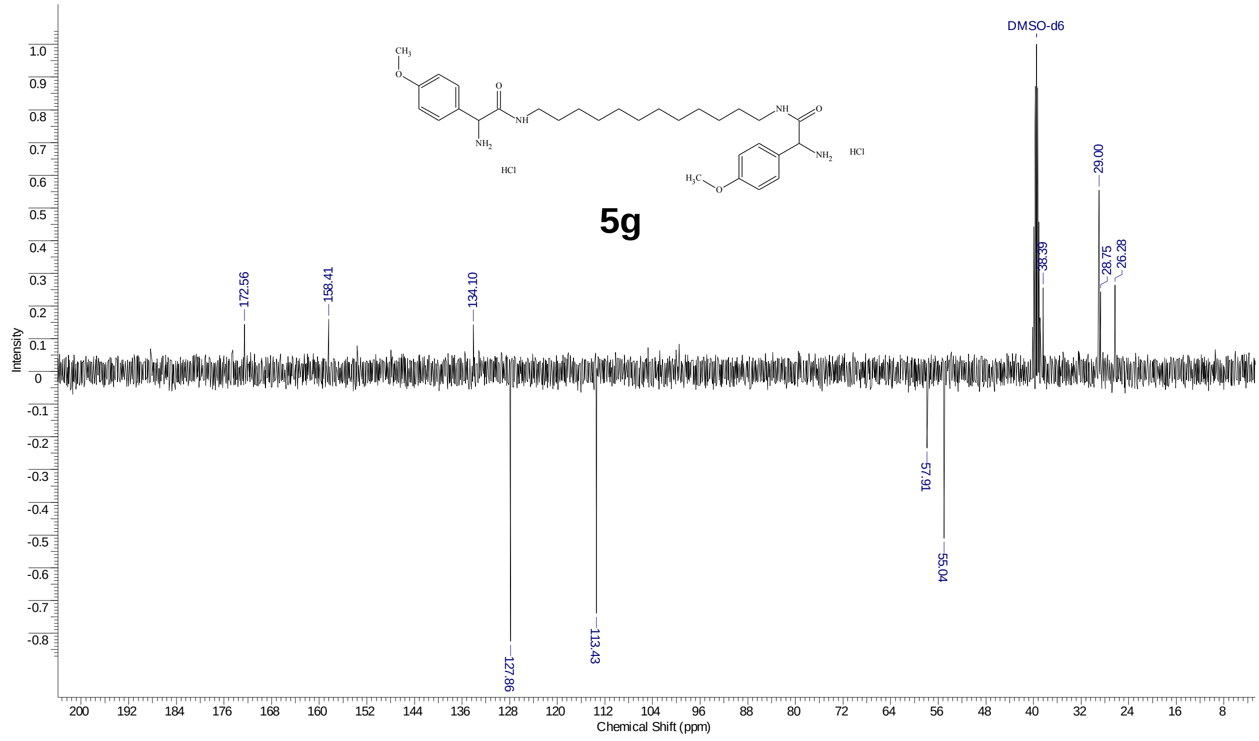
5g
CH3
O
NH
NH2
HCl
H3C
DMSO-d6
172.56
158.41
134.10
127.86
113.43
57.91
55.04
38.39
29.00
28.75
26.28
Intensity
Chemical Shift (ppm)
